# Reaction Mechanism and Kinetic Model of the Transformation of Iron Monosulfide Thin Films into Pyrite Films

**DOI:** 10.1021/acs.jpcc.4c08227

**Published:** 2025-02-19

**Authors:** Carlos Morales, Antonio Pascual, Dietmar Leinen, Gabriel Luna-López, Jose R. Ares, Jan Ingo Flege, Leonardo Soriano, Isabel J. Ferrer, Carlos Sanchez

**Affiliations:** † Applied Physics and Semiconductor Spectroscopy, Brandenburg University of Technology Cottbus–Senftenberg, Konrad-Zuse-Strasse 1, D-03046 Cottbus, Germany; ‡ Dpto. de Física de Materiales, Facultad de Ciencias, Universidad Autónoma de Madrid, Francisco Tomás y Valiente 7, E-28049 Madrid, Spain; § Departamento de Física Aplicada I, Facultad de Ciencias, Universidad de Málaga, Campus Teatinos, C.P. 29071 Málaga, Spain; ∥ Dpto. de Física Aplicada, Facultad de Ciencias, Universidad Autónoma de Madrid, Francisco Tomás y Valiente 7, E-28049 Madrid, Spain; ⊥ Instituto Nicolás Cabrera, Universidad Autónoma de Madrid, Francisco Tomás y Valiente 7, E-28049 Madrid, Spain

## Abstract

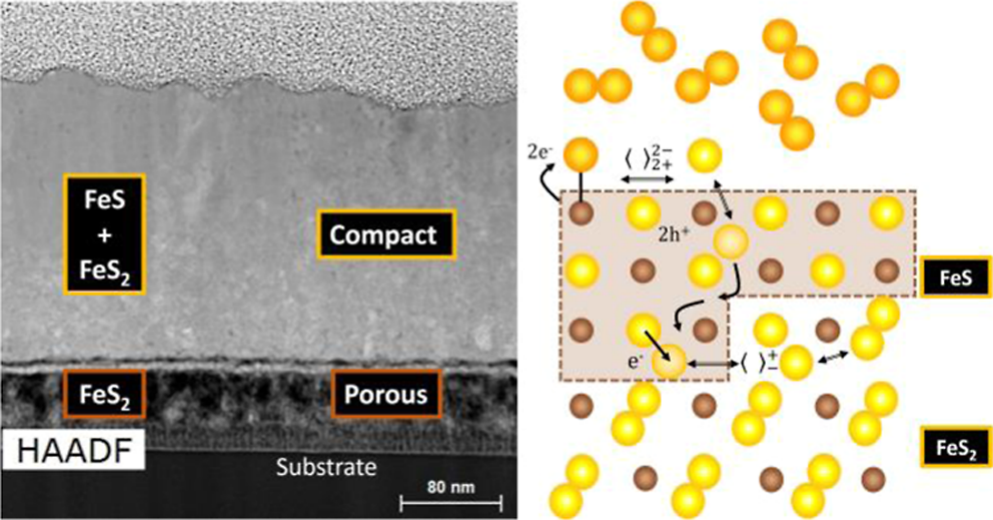

This work presents a comprehensive reaction and kinetic model of the pyrite thin films formation by sulfuration of Fe monosulfides when a molecular sulfur (S_2_) atmosphere is used. This investigation completes the results already published on the explanation and interpretation of the sulfuration process that transforms metallic iron into pyrite. It was previously shown that the monosulfide species (i.e., orthorhombic and hexagonal pyrrhotite phases) are intermediate phases in the sulfuration reaction. Based on experimental data we now show that the sulfuration of pyrrhotite to pyrite takes place in two distinct stages: (i) conversion of orthorhombic pyrrhotite to pyrite (Fe_1–*x*_S^O^ → FeS_2_) while the hexagonal pyrrhotite (Fe_1–*x*_S^H^) phase remains unaltered, and (ii) final transformation of hexagonal pyrrhotite to pyrite (Fe_1–*x*_S^H^ → FeS_2_). Both processes occur via interstitial sulfur diffusion through the previously formed pyrrhotite layer. Consequently, the monosulfide is sulfurated at the internal Fe_1–*x*_S/FeS_2_ interface. The reaction mechanism at each stage has been validated using the corresponding kinetic model to fit the experimental data on time evolution of Fe_1–*x*_S and FeS_2_ layers thicknesses and some of the film transport properties. The concluding global reaction mechanism proposed in some of our former papers and completed here (Fe → Fe_1–*x*_S → FeS_2_) can explain the resulting microstructure of the pyrite films (i.e., Kirkendall effect and formation of a porous layer in the film). Simultaneously, it also justifies the presence of intrinsic defects, such as iron and sulfur vacancies, and the accumulation of interstitial sulfur at the film grain boundaries. The conductivity of pyrite films is tentatively explained using a two-band model where the changes in the Seebeck coefficient and the S/Fe ratio during the pyrite recrystallization stage can be successfully explained.

## 1 Introduction

The ambitious policies toward a fast transition from fossil fuels to a renewable energy system require implementing technologies based on cheap, nontoxic, and abundant elements and compounds.^1^ Consequently, searching for earth-abundant materials for energy-related applications to substitute scarce elements on novel and well-established technologies has become a hot research topic.^2^ In this context, iron sulfides have gained much attention in the past few years thanks to the abundance of iron and sulfur and their low environmental impact.^3^ In particular, pyrite (FeS_2_) is one of the most promising sulfides. In fact, it has been proposed as a photovoltaic material due to its convenient optical bandgap (0.9–1.0 eV)^4,5^ and its high optical absorption coefficient (α ∼ 10^5^ cm^–1^)^4,6^ in a significant part of the solar radiation spectrum. Moreover, the possibility of synthesizing pyrite in different ways (single crystals, thin films, and nanostructures) by different techniques,^7−10^ as well as the use of different dopants to achieve both n- and p-type pyrite films,^11−14^ enables the fabrication of multiple configurations of homo- and heterostructure-based devices.

Despite these advantages and the progress made during the last years, the reduced open circuit photovoltage obtained under solar irradiation (*V*_OC_ ∼ 0.3 V) hinders pyrite photovoltaic applications. Several causes for this limitation have been proposed, as the existence of an inversion layer at the pyrite surface^15,16^ or a high density of deep donor states within the pyrite bulk.^17^ More recently, Voigt and co-workers pointed to the p–n junction at the surface/bulk interface in n-type pyrite single crystals as the potential origin of low *V*_OC_.^18^ Furthermore, there remains a quite open discussion around several surface and bulk characterization topics in both bulk material and thin films. Among them we mention: the role of crystalline defects located at both surface and bulk, i.e., iron and sulfur vacancies;^17,19,20^ the presence of secondary phases at the pyrite grain boundaries^21,22^ or the explanation behind the general p-type behavior of synthetic pyrite films in contrast to the n-type nature of synthetic single crystals.^14,19,23−25^

The macroscopic properties of synthetic pyrite thin films critically depend on the selected growth technique and the applied experimental conditions, which ultimately determine the formation route of pyrite. Due to its simplicity, the so-called sulfuration of iron thin films is one of the most frequently used methods.^26^ This technique enables a high control of several key experimental parameters, such as sulfur partial pressure (*P*_S_2__), sulfuration temperature (*T*_S_), or sulfuration time (*t*_S_). Therefore, slight changes in individual growth factors (while fixing all others) will result in chemical (i.e., stoichiometric) and structural (i.e., crystallization) differences correlated to film macroscopic properties.^27^ For example, variations on *T*_S_ in the ≅350 °C ↔ 400 °C range significantly impact pyrite surface and bulk composition^28^ and crystallite sizes,^29−32^ thus resulting in different macroscopic film properties such as electrical resistivity (ρ)^14,24,27^ or Seebeck coefficient (*S*_th_).^14,25^

Moreover, the mentioned methodology has also allowed for studying the pyrite’s formation mechanism, particularly the appearance of intermediate states. In this respect, Pimenta and Kautek^33−35^ pointed to a faster kinetics for the growth of monosulfide phases (Fe_1–*x*_S) compared to pyrite formation, the first acting as the latter precursor. Furthermore, Pascual^27^ and co-workers arrived at similar conclusions. They showed that the Fe thin film sulfuration implies the initial formation of Fe monosulfides (hexagonal and orthorhombic pyrrhotites), from which the sulfuration process continues toward pyrite. It is important to note that detecting the formed pyrrhotite phases is connected to specific sulfuration conditions, i.e., *P*_S_2__ ≈ 10^–2^ to 10^–1^ Pa, *T*_S_ ≈ 200 °C, and short *t*_S_. These conditions are frequently far from those experimental parameters applied in many studies where only pyrite formation is reported. As a consequence, it is erroneously concluded that iron sulfuration takes place in a single, direct step. Therefore, the present situation calls for a more detailed description of the film behavior during its entire sulfuration route (Fe → Fe_1–*x*_S and then Fe_1–*x*_S → FeS_2_). This improved description could explain the development of chemical and structural defects in the pyrite films as a function of the growth conditions.

We have followed an equivalent procedure to that of our previous works^27,36,37^ consisting of in situ monitoring the thermoelectric properties (i.e., electrical conductivity and Seebeck’s coefficient) of the Fe film during its sulfuration. We recently presented a mechanism and kinetic model regarding the formation of intermediate Fe monosulfide phases at the initial stage of the sulfuration of metallic iron to pyrite thin films.^38^ The diffusion of iron vacancies at the internal Fe_1–*x*_S/Fe interface was identified as the limiting step of the process. Initially, hexagonal pyrrhotite is formed at the film surface via the diffusion of Fe atoms (i.e., Fe vacancies) through the sulfurated (Fe_1–*x*_S) layer. Importantly, this model successfully explained^21,26^ the formation of a porous layer in the internal Fe_1–*x*_S/Fe interface because of the operation of the Kirkendall effect during the monosulfide-phase formation. The formation of that porous layer also justifies why the relative thickness (thickness of the monosulfide film/thickness of the original Fe film) is systematically above the expected values.^22^ Moreover, the conversion from hexagonal to orthorhombic pyrrhotite, both acting as pyrite precursors in the following sulfuration stages,^27^ was shown to follow the dynamics of a Néel transformation.

The present investigation constitutes the natural continuation of the above-mentioned work,^38^ now focusing on the reaction mechanism and kinetic model of the next and final step, i.e., the pyrrhotite transformation into pyrite. Supported by operando thermoelectric measurements (film electrical resistivity, ρ and its Seebeck coefficient, *S*_th_) and ex situ thin film cross-section images taken by transmission electron microscopy and energy-dispersive spectroscopy, we here show how and where the formation of pyrite takes place. Pyrite is formed at the internal FeS/FeS_2_ interface by diffusion of interstitial sulfur, which mainly accumulates at the pyrite film grain boundaries. This step explains the temperature-dependent excess of sulfur in synthetic pyrite thin films grown by this methodology. In our view, the complete understanding and description of the Fe → Fe_1–*x*_S → FeS_2_ sulfuration process will help optimize the growth conditions of pyrite thin films and control the formation of structural, chemical, and stoichiometric defects.

## 2 Experimental Section

Fe metallic films of about 80 nm thickness were deposited by thermal evaporation (Edwards E306 A) of Fe powder (Goodfellow, 99.9%) on soda lima glasses substrates (23 × 9 × 1 mm, Corning 7059). Before metallic Fe deposition, the substrates were washed inside an ultrasonic water bath with neutral soap, rinsed with deionized water, and cleaned with ethanol. Subsequently, substrates were outgassed at 200 °C for 2 h in high vacuum conditions (∼10^–6^ mbar) within the evaporation chamber. Then, the Fe films were deposited on the substrates by thermal evaporation. Immediately after, the obtained Fe thin films were collected from the evaporation chamber and placed in the sample holder of the experimental sulfuration setup.

The complete description of the sulfuration system can be found elsewhere.^38^ Two cylindrical furnaces allow the independent regulation and monitoring of the sample (*T*_sample_) and sulfur source (*T*_sulfur basket_) temperatures. Consequently, precise control of the S_2_ partial pressure, i.e., species responsible for the iron film sulfuration,^39^ is available. A detailed explanation of the S_2_ partial pressure dependence in *T*_sample_ and *T*_sulfur basket_ can be found in the Supporting Information (Table S1 and Figure S1) and our previous work.^38^ During its sulfuration, the sample is placed between two ceramic sheets (30 × 20 × 10 mm) that remain in fixed positions thanks to four screws located at their corners. Thermoelectric measurements are performed through six electrical contacts connected to the exterior by feed-throughs located in the ampule bottom part. Two contacts are used to measure the electrical voltage (*V*_H_ and *V*_C_). Two more contacts will introduce a continuous electrical current (DC+ and DC−). The other two are K-type thermocouples to measure the temperatures of the samplès hot and cold sides (*T*_H_ and *T*_C_, respectively). The sample-furnace geometric configuration creates a thermal gradient through the sample, with a total temperature difference (Δ*T* = *T*_H_ – *T*_c_) of about 3–4 °C between its ends. This configuration allows to perform in situ measurement of the film Seebeck coefficient (*S*_th_) and electrical resistance (*R*) during its sulfuration process. First, *S*_th_ is obtained as the quotient of the thermoelectric voltage and the temperature difference (*S*_th_ ≈ (*V*_C_ – *V*_H_)/(*T*_H_ – *T*_C_)). We assume the Telkes criterion about the sign of the *S*_th_ coefficient.^40^ The film electrical resistance is measured by the four probes method: a continuous electrical current (*I*) is applied through contacts DC(+) and DC(−), and then the voltage difference Δ*V* = *V*_C_^′^ – *V*_H_^′^ is measured. As the temperature difference increases, however, it simultaneously establishes a Seebeck’s potential Δ*V*_S_ = *V*_C_ – *V*_H_, and thus the real ohmic voltage difference must be corrected: Δ*V* = (*V*_C_^′^ – *V*_H_^′^) ± |Δ*V*_S_| (Δ*V*_S_ may be positive or negative depending on the n- or p-type nature of the sample). The minimum pause between two complete sets of measurements (*R* and *S*) is 5 s. More details about the experimental setup can be found elsewhere.^27,36,37^

Complementary ex situ measurements were taken at room temperature after stopping the film sulfuration process at the desired sulfuration stage and cooling the sample. X-ray diffractograms (XRD) were done with an X’Pert PRO θ/2θ Panalytical diffractometer in grazing incidence configuration using Cu Kα radiation (Cu Kα = 1.5406 Å) and an incidence angle of 1.7°. The cross-section morphology of the samples was studied by transmission electron microscopy (TEM) images obtained using a FEI Talos F200X equipped with a FEG electron source of 200 keV and a minimum resolution of 0.16 nm. Lamellas of the films for electron transmission analysis were prepared within an SEM Dual Beam Helios NanoLab 650 (FEI) system. To that end, a Tomahawk TM ion source of gallium for focused ion beam (FIB) manipulation was used. Compositional energy dispersive X-ray (EDX) analysis along the film cross sections was performed with this same instrument. Besides, S/Fe atomic ratios were determined in different points of the films by EDX analyses with an Oxford software associated with a scanning electron microscope (SEM, Hitachi S-3000 N). All the EDX data were obtained with an electron beam energy of 15 keV under normal incidence to the sample surface. Film thicknesses before and after their sulfuration were measured by profilometry (Sloan Dektak IIA Profilometer, accuracy ±1 nm). Finally, ex situ RT resistivity (ρ) and Seebeck’s coefficient (*S*_th_) measurements of the sulfurated films were also done through the Van der Pauw method and equipment designed in our lab,^41^ respectively.

## 3 Results and Discussion

The Fe → Fe_1–*x*_S → FeS_2_ sulfuration process has been qualitatively described by Pascual et al.^27^ as taking place in four differentiated stages. The first one corresponds to the complete sulfuration of iron into monosulfides. First via the transformation of metallic iron to hexagonal pyrrhotite (Fe → Fe_1–*x*_S^H^) and then by a partial conversion of the formed pyrrhotite to orthorhombic one (Fe_1–*x*_S^H^ →Fe_1–*x*_S^O^). A comprehensive description of the reaction mechanism and kinetic model of these two stages was recently published by our group.^38^ The next two stages consist of the sulfuration of the pyrrhotite phases toward pyrite. This sulfuration takes place in two equivalent chemical steps: sulfuration of orthorhombic pyrrhotite to pyrite (Fe_1–*x*_S^H^ + Fe_1–*x*_S^O^ → Fe_1–*x*_S^H^ + FeS_2_) and, subsequently, sulfuration of hexagonal pyrrhotite to pyrite (Fe_1–*x*_S^H^ + FeS_2_→ FeS_2_). The fourth final stage corresponds to the crystallization of the already formed pyrite film. A series of in situ thermoelectric measurements and ex situ XRD diffractograms of selected samples have confirmed the differentiation and reproducibility of all stages. To this purpose, the sulfuration process was quenched at different critical moments (see Figure S2)^27,36−38^ to obtain the convenient samples. The presence and relative amount of FeS_1–*x*_ and FeS_2_ phases at each moment can be inferred from XRD and EDX measurements. However, a certain degree of uncertainty is present due to possible poor crystallization of the films and excess/defect of sulfur at some stage.

This way, consecutive samples B, C, D, and E (indicated in Figure S2) corresponding to stages 2 and 3 of the process were selected (global Fe_1–*x*_S → FeS_2_ transformation). As previously mentioned, the sample obtained at the starting point (B) consists of a pyrrhotite film (atomic S/Fe ratio of 1.14, i.e., Fe_0.88_S) with two phases, Fe_1–*x*_S^H^ (∼42%) and Fe_1–*x*_S^O^ (∼58%). The relative amount of each phase was calculated using the relative intensity of their respective diffraction peaks (see Table S2 and Figure S3 for the crystallographic details). To this end, it was assumed that both phases have similar densities (∼4.5–4.7 g/cm^3^) and stoichiometries (S/Fe ∼ 1.05–1.14).^38,42^ Furthermore, samples C and D correspond, respectively, to the intermediate and final states of stage 2, where the orthorhombic phase is wholly transformed into pyrite while the amount of the hexagonal phase remains almost constant. In this case, sample C is composed of Fe_1–*x*_S^O^ (∼18%), Fe_1–*x*_S^H^ (∼38%), and pyrite (∼44%), whereas sample D is formed only by Fe_1–*x*_S^H^ (∼41%) and pyrite (∼59%). It is not until stage 3 of the sulfuration process that the formation of FeS_2_ is completed (sample E). The corresponding film has an atomic S/Fe ratio of about 2 and mainly pyrite contributions in its XRD pattern, with tiny amounts of marcasite present. However, the contribution of small fractions of amorphous pyrrhotite phases cannot be discarded entirely at this stage. The orthorhombic and hexagonal pyrrhotite phases have very similar stoichiometry and density but differ in their crystallographic structure and electrical properties (resistivity and Seebeck coefficient).^37,38^ Therefore, the molecular model of pyrite formation from both pyrrhotite phases should be quite similar. However, the limiting step of the processes could change as a function of *t*_S_ and *P*_S_2__, and thus, it could be different for stages 2 and 3.

Further recrystallization of pyrite (stage 4) greatly depends on *T*_S_ and *t*_S_, and it may induce changes in the S/Fe atomic ratio and increase the crystallite size^28,29,32^ and film thickness.^28,30,31,43^ Under the sulfuration conditions used in this work, *T*_S_ ∼ 330 °C and *t*_S_ ∼ 30 h (almost 20 h during stage 4), the crystallization of pyrite takes place, as comprehensively described in our previous works.^22,27,28,32^Table 1 summarizes the four stages of the complete sulfuration process and the associated model samples and figures presented in the present work. It is worth noting that using SLG substrates promotes the diffusion of Na from the substrate into the film during the recrystallization stage (the diffusion starts once the pyrrhotite is transformed into pyrite).^28^ At moderate sulfuration temperatures (*T*_S_ < 400 °C), the diffusion of Na occurs through the pyrite grain boundaries, accumulating there and mainly in the internal porous layer provoked by the Kirkendall’s effect during stage 1.^22^ However, this accumulation seems to have no apparent influence on the film thermoelectric properties such as the Seebeck coefficient. The doping of pyrite with Na seems to be only relevant at sulfuration temperatures higher than those used in this work. In these conditions, the Na atoms seem to diffuse through the pyrite grains, probably occupying Fe-vacancies (V_Fe_) sites, and accordingly^14,28^ decreasing the positive Seebeck coefficient. Similar changes in the electrical resistivity and Seebeck and Hall coefficients of n-type doped (Al, Ti, Co, Ni, Cu) pyrite thin films as a function of *T*_S_ have already been reported.^13,14,44−47^

**Table 1:** Summary of the Reaction Stages of the Complete Sulfuration Process Fe → FeS → FeS_2_

stage	sulfuration step	samples and figures	composition
stage 1	(1) Fe → FeS^H^ + FeS^O^	sample A^27,38^	Fe
	(2) (partial) FeS^H^ → FeS^O^	sample B^38^	FeS^H^ (∼42%), FeS^O^ (∼58%)
stage 2	FeS^O^ → FeS_2_ (pyrite)	sample C Figures 1, 4, and 5	FeS^H^ (∼38%), FeS^O^ (∼18%), pyrite (∼44%)
stage 3	FeS^H^ → FeS_2_ (pyrite)	sample D	FeS^H^ (∼41%), pyrite (∼59%)
		sample E Figures 1, 6, and 7	FeS_2_ (pyrite)
stage 4	FeS_2_ recrystallization	Figure 8	FeS_2_ (pyrite)

Considering these experimental facts, we will thoroughly focus on the reaction mechanism and kinetic model of stages (2) and (3) of the sulfuration process in the following sections. We have followed a similar approach to that in ref (38) and, thus, completed the picture of the whole sulfuration process. Afterward, the model will be applied to discuss the formation of local defects in pyrite thin films and their influence on some macroscopic properties, such as electrical resistivity and Seebeck’s coefficient.

### 3.1 Pyrrhotite Sulfuration into Pyrite: Reaction and Kinetic Model

The transformation of iron monosulfide into disulfide implies the necessary incorporation of one sulfur atom into the pyrrhotite lattice, as shown by
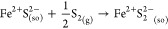
where the subindex (g) and (so) refer to gas and solid phases, respectively. The Fe–S bonds in the pyrrhotite and pyrite molecules are different. While the bond is double ionic in the monosulfide (the sulfur atom takes two electrons from the iron atom), pyrite presents a single ionic bond between each sulfur and the iron atoms and a covalent bond between the two sulfur atoms. Therefore, the oxidation state of sulfur changes from double to single-ionized atoms. In order to form a molecule of pyrite from a pyrrhotite molecule, one of the ionic bonds of the latter must be broken. Then, the electron resulting from it must pass to the newly incorporated sulfur atom so that the two sulfur atoms will form a covalent bond between them.

**Figure 1:**
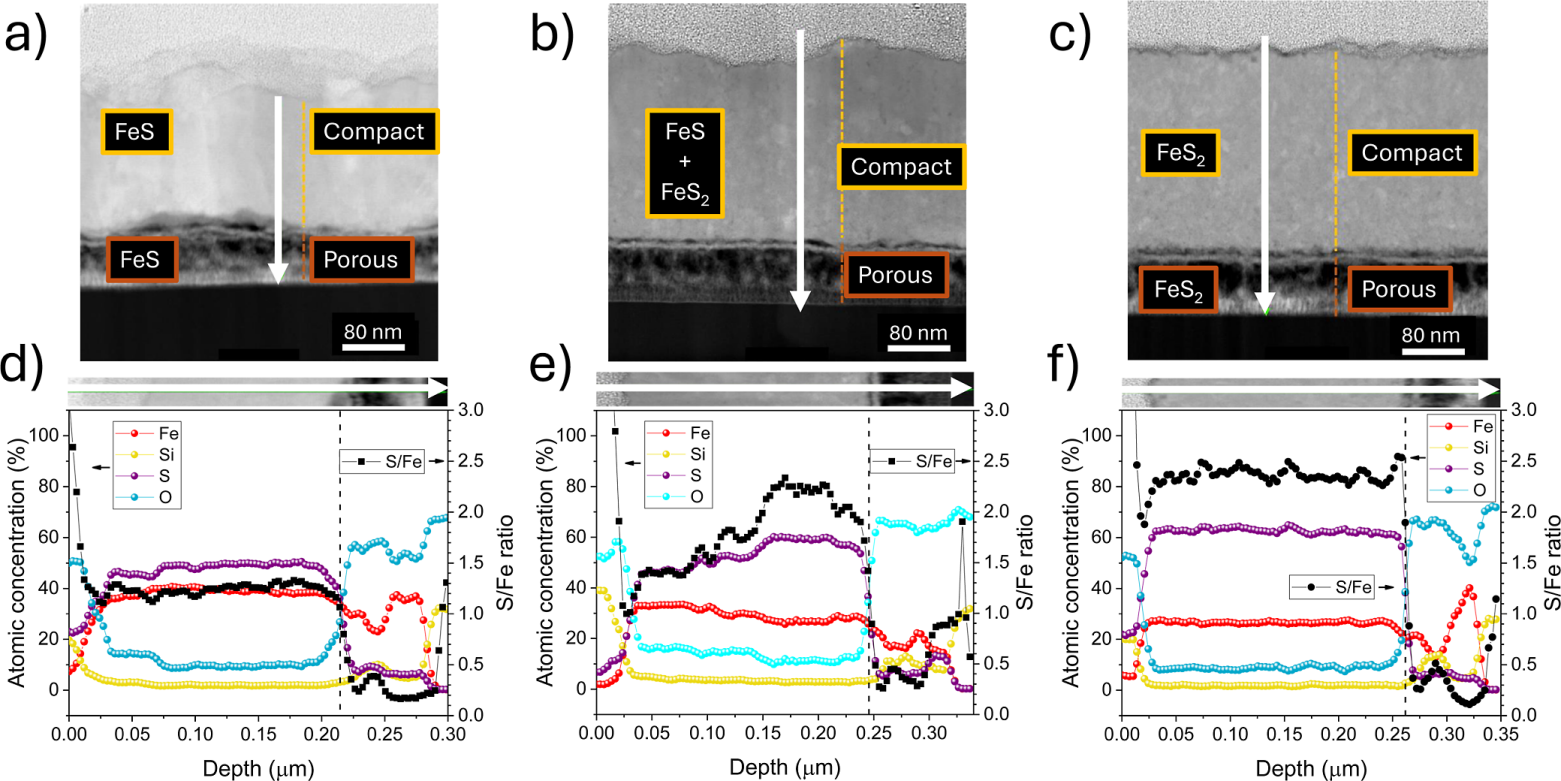
From left to right in the top panel: cross-section TEM-HAADF image of sulfurated thin films at equivalent conditions to those applied to samples (a) B, (b) D, and (c) E in Figure S2. The white arrows indicate the position of the EDX linear scan performed across the thin film. In the bottom panel and following the same order, figures (d–f) depict the atomic concentration (left *y*-axis) and the atomic S/Fe ratio (right *y*-axis) as a function of depth inferred from the linear EDX scan of figures (a–c), respectively.

Figure 1 shows the TEM cross-section of sulfurated samples equivalent to B, D, and E in Figure S2 (detailed elemental EDX mappings of sample C are shown in Figure S4). As can be observed in Figure 1 from the EDX mappings and selected line scans, pyrite formation occurs from the bottom to the top of the film. This fact implies that the dissociation of the sulfur S_2_ molecule takes place at the pyrrhotite surface. This step is followed by the subsequent diffusion of sulfur through the pyrrhotite matrix until the sulfuration reaction takes place at the internal interface between the Fe_1–*x*_S and FeS_2_. Therefore, once stage 1 (Fe → Fe_1–*x*_S) ends after the complete depletion of the metallic iron film, the sulfur atoms adsorbed at the pyrrhotite surface start to diffuse through the pyrrhotite compact layer. Then, they accumulate at the porous layer, a phenomenon previously observed by TEM cross-section images in similar sulfurated samples.^22^ The sulfur accumulation induces the nucleation of the thermodynamically more stable pyrite phase at the internal interface.^33^ From there, the pyrite compact layer continues growing, fed by the newly diffused S atoms. The structure of the porous layer, which corresponds to the voids left by the diffusion of Fe atoms from the metallic layer during stage 1, does not change during stages 2 and 3. However, it presents a higher oxygen concentration, likely diffused from the SLG substrate.^14,22,38^

**Figure 2:**
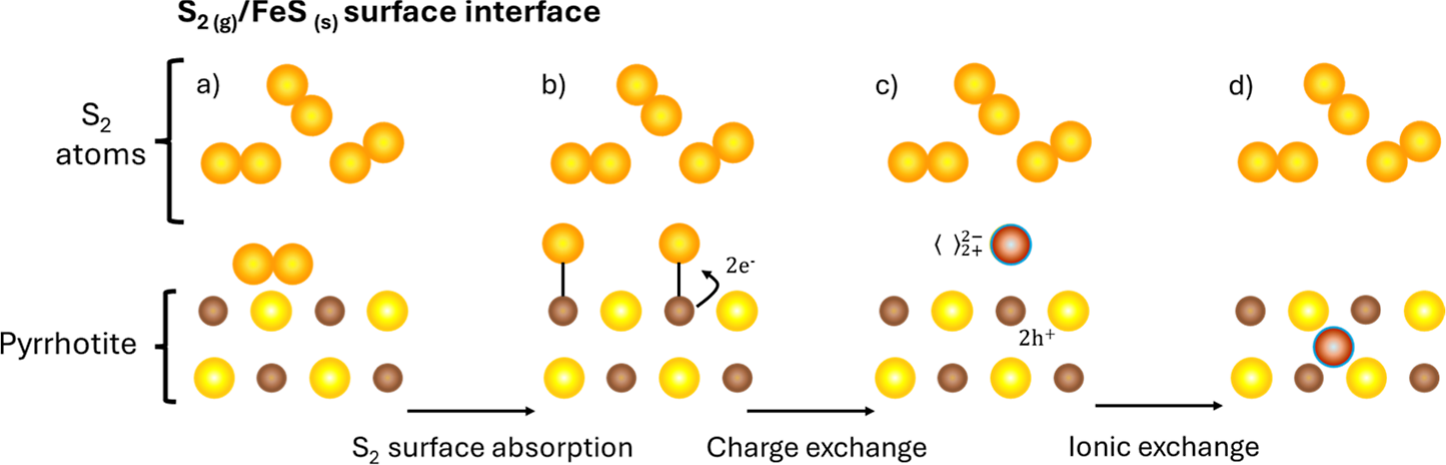
Schematic representation of the Fe_1–*x*_S → FeS_2_ sulfuration process at the sample external S_2_/pyrrhotite interface. From left to right, the sketches represent: (a,b) sulfur adsorption on pyrrhotite surface, and charge (b,c) and ionic (c,d) exchange steps of the sulfur/phyrrhotite reaction at the external interface. The Fe and S atoms in the Fe_1–*x*_S matrix and S atoms from S_2_ and interstitial species are represented by brown, yellow, orange, and dark-orange spheres, respectively. The transformation from pyrrhotite to pyrite at the internal pyrrhotite/pyrite interface is considered in Figure 3.

**Figure 3:**
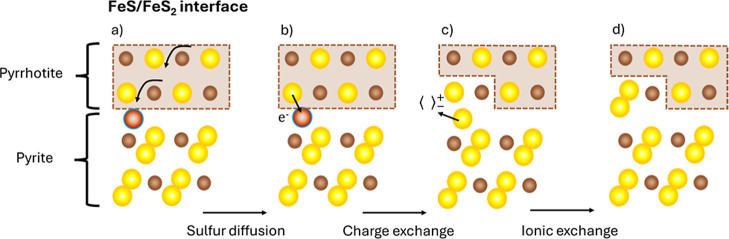
Schematic representation of the Fe_1–*x*_S → FeS_2_ transformation process at the internal pyrrhotite/pyrite interface during sulfuration. From left to right, the sketches represent: (a) sulfur diffusion toward the pyrrhotite phase, and charge (b,c) and ionic (c,d) exchange steps of the phyrrhotite/pyrite reaction at the internal interface. The Fe and S atoms from Fe_1–*x*_S and FeS_2_ matrix are represented by brown and yellow spheres, respectively. The interstitial species are represented by dark-orange spheres. The pyrrhotite phase appears enclosed in a brown box.

As in stage 1 (Fe → Fe_1–*x*_S^H^),^38^ the following reaction is heterogeneous and can be divided into four steps. These steps are (I) sulfur adsorption at the external pyrrhotite surface; (II) reaction sulfur–pyrrhotite at the external film interface; (III) diffusion of interstitial sulfur through the formed pyrrhotite phase layer; and (IV) reaction at the internal interface (pyrrhotite–pyrite). Eyring’s theory of absolute velocities can be applied to each elementary reaction to obtain their corresponding rates.^48^Figures 2 and 3 show a schematic and qualitative representation of the complete sulfuration process of pyrrhotite at the external (I, II, and III steps) and internal interfaces (IV step), respectively. As step IV occurs at the pyrrhotite/pyrite interface, we assume the ultrafast nucleation of an initial ultrathin layer of pyrite promoted by sulfur accumulation at the porous region. As in our previous work,^38^ we will continue using Besson’s notation in the following (a detailed explanation of the meaning of the different symbols can be found in the Supporting Information).

#### 3.1.1 Sulfur Adsorption on Pyrrhotite Surface

The S_2_ molecule dissociates at the pyrrhotite surface through a physisorption process consisting of a partial electron transfer from the metallic cation to the gas atom
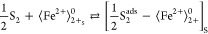
I

The subscript s indicates that the reaction occurs at the surface, and the right-hand square bracket refers to the described intermediate species (see Figure 2a,b). The corresponding kinetic equation and associated adsorption velocity, *v*_a_, can be expressed in terms of the total fraction of occupied active surface sites as a function of time, θ(*t*). The velocity *v*_a_ will also depend on the partial pressure of S_2_ species
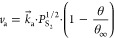
1where  is the kinetic constant of the direct reaction. More details can be found elsewhere^38^ and in the Supporting Information.

#### 3.1.2 Reaction (Sulfur–Pyrrhotite) at the Film External Interface

This reaction takes place in two steps. On the one hand, the charge exchange step (II.a) is the same as during stage 1: the pyrrhotite Fe atom transfers two electrons to the adsorbed sulfur atom. In this way, it becomes part of the monosulfide lattice together with the formation of a cation vacancy (Figure 2b,c). Two holes weakly bound to the Fe atom at interstitial sites result from this charge exchange
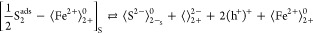
II.a

The corresponding kinetic equation is
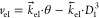
2where *D*_1_ is the concentration of the cation vacancies and  and  are the kinetic constants for the direct and inverse reactions.

The second step corresponds to the ionic exchange (Figure 2c,d): the sulfur atom that had become part of the pyrrhotite lattice moves to the interstitial occupied by two holes, giving rise to an interstitial neutral sulfur atom
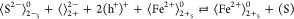
II.b

It is important to note that not all the holes that are created during step II.a (*D*_1_) will be assimilated in II.b. This implies that the concentration of interstitial atoms (*C*_e_) will be lower than in other conditions (we will return to this point when discussing the generation of defects in pyrite thin films and their influence on the thermoelectric coefficient). Therefore, the kinetic equation results
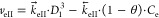
3where  and  are the kinetic constants for the direct and inverse reactions, respectively. Equations 2 and 3 can be combined by assuming stationary conditions (i.e., ), which defines the concentration of interstitial sulfur atoms under equilibrium conditions, *C*_e_^∞^
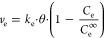
4

#### 3.1.3 Diffusion of Interstitial Sulfur through the Pyrrhotite Phase

The concentration gradient of the sulfur atoms between the external (sulfur/pyrrohotite) and internal (pyrrhotite/pyrite) interfaces controls their diffusion through the pyrrhotite layer, as illustrated in Figure 3a. If *C*_i_ is the concentration of sulfur atoms at the internal interface, then the equation describing the diffusion process of sulfur atoms can be written as
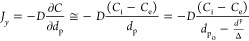
5where *D* is the diffusion coefficient of the sulfur atoms in pyrrhotite, *d*_p_ is the pyrrhotite layer thickness, *d*_po_ the initial pyrrhotite thickness at the beginning of stage 2, and *d*^P^ is the pyrite thickness corrected by the factor Δ to account for the change in the unit cell volume.^22,38^

#### 3.1.4 Reaction at the Internal Interface (Pyrrhotite–Pyrite) of the Film

As for the external interface reaction, we can divide the pyrrhotite/pyrite reaction into two steps. First, the charge exchange (III.a, see Figure 3b,c) implies breaking the pyrrhotite ionic bond by transferring one electron from the double-ionized sulfur anion to the interstitial sulfur. This sulfur anion becomes a pyrite single-ionized anion at an interstitial site, while the pyrrhotite molecule transforms into a pyrite one with a sulfur vacancy
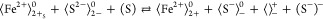
III.a

The reaction will be controlled by the concentrations, at the internal interface, of interstitial sulfur atoms (*C*_i_), pyrrhotite molecules at a given time (*E*_*t*_), and of the interstitial anions and sulfur vacancies that are formed (*E*_1_). Therefore, by considering the kinetics constants  and , the reaction velocity can be obtained
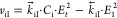
6

Finally, the second step consists of the ionic exchange reaction (III.b), see (Figure 3c,d), where the interstitial anion passes to occupy the position of the sulfur vacancy, thus completing the formation of the pyrite molecule
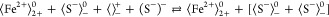
III.b

Similarly to the previous step (III.a), we define *E*_o_ as the maximum concentration of pyrrhotite molecules at the internal interface and apply the theory of absolute velocities
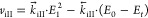
7where  and  are the kinetics constants, respectively. Similarly to the reaction at the sulfur/pyrrhotite external interface, expressions6 and 7 can be combined by considering stationary conditions, i.e., under equilibrium, where *C*_i_^∞^ is the concentration of interstitial sulfur atoms at the internal interface in these conditions
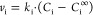
8

The first column of Table 2 summarizes the velocity expressions obtained for the four elementary reactions involved in the pyrrhotite sulfuration into pyrite (1, 4, 5, 8). Interestingly, all of them have turned out to be similar to those describing the transformation of metallic Fe to hexagonal pyrrhotite (stage 1).^38^ Therefore, it seems that the form of these expressions depends, basically, on the type of conductivity that generates the created lattice defect that, subsequently, diffuses.^49,50^ In other words, the expressions are similar regardless of whether the sulfuration takes place via formation and diffusion of Fe vacancies (stage 1) or by sulfur interstitials (stages 2 and 3) since, as has been shown, holes are created in both cases (p-type conductivity in the material). The reaction constants, diffusion coefficient, and concentrations (both at and away from equilibrium) have different values in both Fe → Fe_1–*x*_S, and Fe_1–*x*_S → FeS_2_ processes. Furthermore, although the transformations of orthorhombic and hexagonal pyrrhotite phases into pyrite are chemically equivalent, and thus, the same expressions for the elementary reaction velocities can be used, the limiting process will depend on the experimental conditions.

**Table 2:** Summary of the Kinetic Equations of the Elemental Reactions Involved in the Sulfuration Process FeS → FeS_2_^a^

elemental reaction	kinetic equations	reaction velocities as a function of *P*_S_2__	evolution of pyrite thickness
(I) absorption	(1, T1)	(T5)	(T9)
(II) reaction at the external interface	(4, T2)	(T6)	(T10)
(III) diffusion	(5, T3)	(T7)	(T11)
(IV) reaction at the internal interface	(8, T4)	(T8)	(T12)

a The evolution of pyrite thickness has been calculated using (9) by integrating expressions T5–T8 and using *P*_S_2__ = °*P*_S_2__·e^*t*/τ^.

The next sections will discuss stages 2 and 3 of the whole process following the same steps: (i) determination of the limiting process (i.e., limiting velocity), (ii) calculation of the time evolution of Fe_1–*x*_S^O^, Fe_1–*x*_S^H^, and FeS_2_ layers thicknesses by considering the kinetic model, and (iii) validation of the kinetic model by fitting the experimental *S*_th_ and R in situ transport measurements by using the thickness evolution estimated in (ii).

### 3.2 Stage 2: Fe_1–*x*_S^O^ → FeS_2_

#### 3.2.1 Limiting Velocities as a Function of the Experimental Parameters for Fe_1–*x*_S^O^ → FeS_2_: Evolution of the Film Thickness

**Figure 4:**
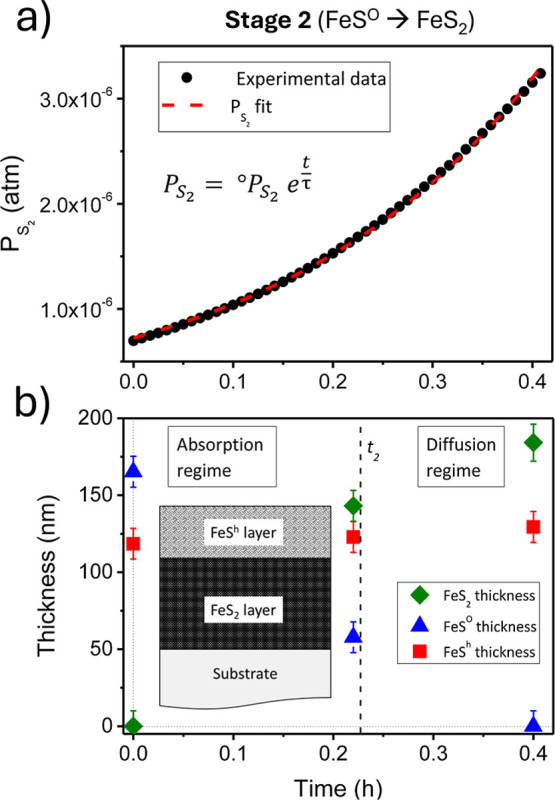
(a) Time-evolution of S_2_ partial pressure during stage 2 of the sulfuration process from monosulfide to pyrite, i.e., FeS^O^ to FeS_2_ step. (b) Estimated thicknesses of sample B (beginning stage 2, thin film composed by a mixture of Fe_1–*x*_S^O^ and Fe_1–*x*_S^h^ pyrrhotite phases); sample C (close to *t*_2_ time, mixture of FeS_2_, Fe_1–*x*_S^O^, and Fe_1–*x*_S^h^) and sample D (end of stage 2, thin film formed by FeS_2_ and Fe_1–*x*_S^h^), as depicted in Figure S2. The dashed line indicates the time (*t*_2_) when the change from the absorption to diffusion regimes occurs. The bottom insert provides a sketch of the thin film cross-section at this stage. Profilometry has been used to determine the total thickness and XRD data to obtain the relative thickness of each phase (FeS_2_, Fe_1–*x*_S^O^ and Fe_1–*x*_S^h^).

The experimental conditions at which stage 2 occurs are depicted in the Supporting Information, Figure S5. Whereas the temperature of the sample remains constant during the entire stage, the temperature of the sulfur basket increases at a constant rate. This step translates into an increase in the total sulfur pressure with the corresponding *P*_S_2__. Figure 4a shows the time evolution of *P*_S_2__ during stage 2. These data can be fitted to an exponential expression in the form of *P*_S_2__ = °PS__2__e^*t*/τ^. The reaction velocities (2, 5, 6, and 8) can be expressed as a function of the “dissociation pressure” of pyrite (°*P*_S_2__) and the partial pressure of S_2_ species, *P*_S_2__. This parameter constitutes the only measurable experimental condition that directly influences the velocity of the reactions. These expressions are shown in the second column of Table 2 (T5–T8). Furthermore, the time evolution of pyrite film thickness, *d*^P^, is obtained from the following expression
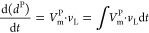
9where *V*_m_^P^ is the molecular volume of pyrite (considered constant during the complete Fe_1–*x*_S → FeS_2_ transformation), and *v*_L_ is the velocity of the limiting process. The third column of Table 2 shows the resulting time evolution of pyrite thickness for each one of the elementary reactions as a function of *P*_S_2__ (T9–T12).

To our knowledge, however, all works dealing with oxidation or sulfuration of metal monoxides and monosulfides accept that the reactions at the equivalent external or internal interfaces are very fast. This is due to the high affinity that such compounds usually show toward the respective gases. Therefore, the growths are controlled by gas surface adsorption or diffusion.^51−56^ By considering this assumption, let first discuss qualitatively which would be the limiting process in stage 2. If a diffusion-controlled growth expression depending on *P*_S_2__ is accepted (T11), it is not possible to fit the obtained experimental data of stage 2 (see Figure 4b). A second possibility would be that the gas diffusion is independent of the S_2_ partial pressure, i.e., a Wagner-type diffusion process
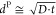
10

For stage 2 to begin, however, it is necessary to increase the partial pressure of S_2_ in the experimental system in relation to that at the end of stage I.^27,37,38^ This step suggests that this pressure must play an important role in the pyrite formation process. The whole Fe_1–*x*_S^O^ → FeS_2_ sulfuration process is not fitted by the expression obtained when the growth is controlled only by the surface adsorption reaction of S_2_ (T9). Therefore, we propose that the overall process is initially controlled by the surface adsorption reaction of S_2_ up to a time *t*_2_. After that, the diffusion process becomes slower and controls the pyrite formation during the rest of the stage. This time *t*_2_ would correspond to a kink in Seebeck’s coefficient time evolution for the in situ measurements (see Figure S2 and Pascual et al.^27^), at which time sample C sulfuration is quenched. As in the previous case (complete diffusion control), to obtain a good fit of the experimental data it is necessary to accept that the diffusion from time *t*_2_ onward does not depend on the pressure of S_2_. This assumption imposes a Wagner diffusion mechanism. Therefore, the pyrite thickness evolution is given by
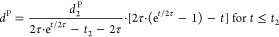
11
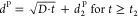
12

**Figure 5:**
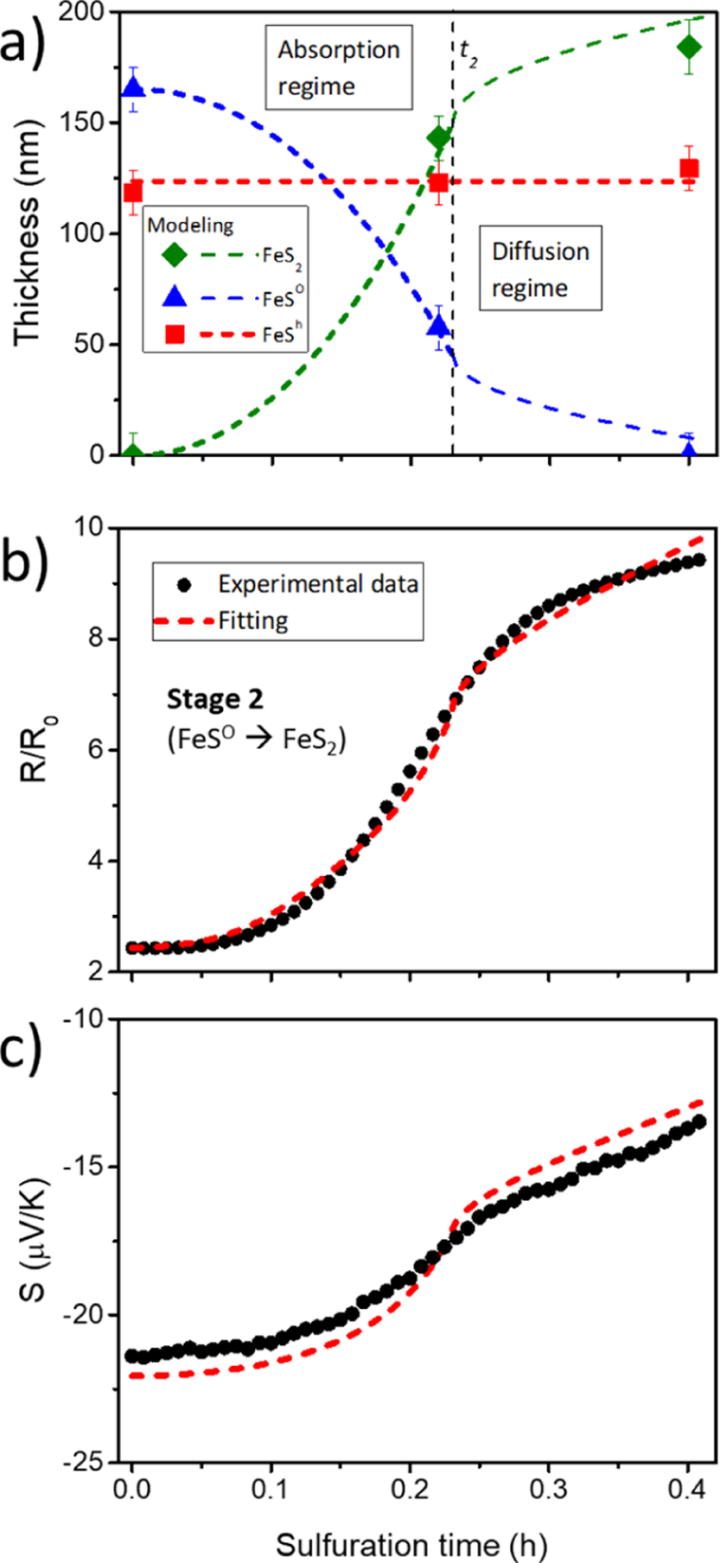
(a) Thicknesses evolution of the pyrrhotite Fe_1–*x*_S^O^ and Fe_1–*x*_S^h^ phases and FeS_2_ layer with the sulfuration time as extracted from the applied model, eqs 11 and 12. The black, vertical dashed line indicates the time (*t*_2_) when the change from the absorption to diffusion regimes occurs. The experimental data (full symbols) correspond to those shown in Figure 4b. (b) Electrical resistance of the sample (*R*) normalized to the Fe film resistance at RT (Ro) and (c) Seebeck’s coefficient (*S*) of the films as a function of the sulfuration time. The black circles represent the experimental data obtained from sample D during its sulfuration process, while the red dashed lines show the fitted values obtained according to expressions13 and 14 by using the thickness evolution plotted in (a).

Figure 5a shows the thickness-time evolution of the pyrite and orthorhombic pyrrhotite layers using expressions11 and 12 and the corresponding experimental values (time *t* = 0 h corresponds to the starting point of stage 2). Recapitulating, we have considered that the process is initially (up to ∼ 0.23 h) controlled by the sulfur surface adsorption. Then, a Wagner diffusion of interstitial sulfur atoms becomes the limiting process until the complete disappearance of the orthorhombic monosulfide. The thickness of the hexagonal pyrrhotite phase was considered constant during stage 2. The kinetic model fits the experimental data well. The obtained diffusion coefficient of sulfur in pyrrhotite is *D* ∼ 3.5 × 10^–14^ cm^2^/s (*T* ∼ 600 K). This value is between those reported by Condit et al. (∼10^–9^ to 10^–12^ cm^2^/s) at higher temperatures (*T* ∼ 1200 K) in monosulfide crystals^57^ and that reported by Pimienta and Kautek in sulfurated Fe thin films^33^ (10^–17^ cm^2^/s at ∼600 K). The difference could be related to a relatively poor crystallization of our Fe_1–*x*_S^O^ layer due to the low sample temperature in stages 1 and 2.

#### 3.2.2 Modeling of the Reaction: *R* and *S*_th_ as a Function of the Sulfuration Time and Comparison with Experimental Results

As described in our previous work,^38^ the thin film thickness-time evolution can be used to fit the in situ experimental data of the film thermoelectric coefficient (*S*_th_) and electrical resistance (*R*). For this purpose, the sample cross-section morphology is simplified by considering that it is formed by three parallel layers whose thicknesses evolve with the sulfuration time. During this time, the electrical resistivities (ρ) and Seebeck coefficients of pure Fe_1–*x*_S^O^, Fe_1–*x*_S^H,^ and FeS_2_ are considered constants (see Supporting Information for more details). This approximation of the sample geometry is reasonable, as depicted by the TEM cross-section images in Figure 1. Therefore, equivalent electrical circuits can be inferred using this sample geometry (see Figure S8), obtaining the following expressions for *R* and *S*
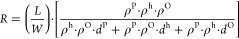
13
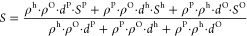
14where superscripts P, h, and O indicate pyrite, hexagonal, and orthorhombic pyrrhotites, respectively. Also, ρ, *S*, and *d* refer to the resistivity, Seebeck coefficient, and thickness of the corresponding phase. The  geometric factor, where *L* and *W* are the dimensions of the sample, relates the two electrical measurements (*R* and *S*) carried out with different contact geometries. As explained in the Supporting Information and plotted in Figure S10, a sigmoid-type function has been used to account for the geometric factor time evolution.

Figure 5b (*R*/*R*_o_) and 5c (*S*_th_) show the excellent reproducibility of the experimental data. The calculated values are within a 10% error compared to experimental data. This indicates that the approximations made (particularly the one referred to constant values of ρ and *S*_th_ for each phase independently of their thickness and potential structural changes) are acceptable. It is worth mentioning that a Wagner-type controlled diffusion for the whole stage 2 cannot fit the data correctly, as shown in Figure S12. Therefore, these results confirm that the sulfuration process during stage 2 can be divided into two regimes: a first one controlled by the S_2_ adsorption at the surface; and, second, a Wagner-type diffusion of sulfur once a sufficient concentration of adsorbed sulfur at the surface is reached.

### 3.3 Stage 3: Fe_1–*x*_S^H^ → FeS_2_

#### 3.3.1 Limiting Velocities as a Function of the Experimental Parameters for Fe_1–*x*_S^H^ → FeS_2_: Evolution of Thickness

**Figure 6:**
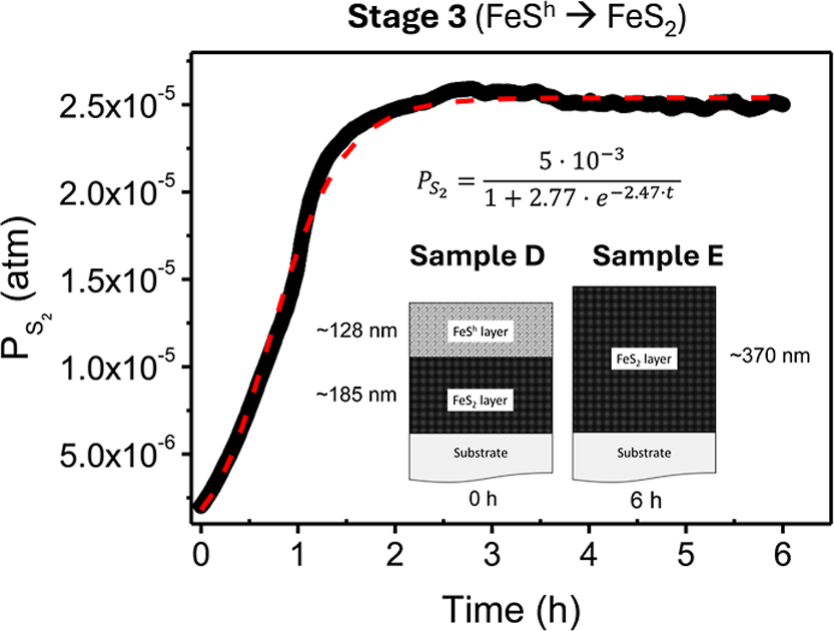
Time-evolution of S_2_ species partial pressure during stage 3 of the sulfuration process (from hexagonal pyrrhotite to pyrite, i.e., Fe_1–*x*_S^h^ to FeS_2_ step). The inset provides a sketch of the thin film cross-section of samples D and E of Figure S2, i.e., at the beginning and end of stage 3. Profilometry has been used to determine the total thickness and XRD data to obtain the relative thickness of each phase (FeS_2_ and Fe_1–*x*_S^h^).

During stage 3, the sample temperature remains essentially constant while the partial pressure of S_2_ species stabilizes at its maximum value (see Figure S6). For completeness, the fit of S_2_ with time is plotted in Figure 6. However, we do not expect the S_2_ partial pressure to control stage 3. This view is supported by (i) stage 2 ends controlled by a Wagner-type diffusion process, and (ii) the S_2_ partial pressure continues increasing during stage 3. Therefore, we can rule out that the transformation of hexagonal pyrrhotite to pyrite is governed by a S_2_ adsorption reaction. By the same arguments, we disregard that a pressure-dependent diffusion mechanism, or a pressure-dependent process at the external or internal interfaces, governs the transformation of hexagonal pyrrhotite to pyrite. For example, if the process were controlled by the reaction at the internal pyrrhotite/pyrite interface dependent on S_2_ partial pressure, the obtained thickness could not fit the experimental values (see Figure S13a).

If a Wagner-type diffusion process with a constant diffusion coefficient (as in stage 2) is considered, the obtained thickness could neither perfectly fit the evolution of Fe_1–*x*_S^H^ and FeS_2_ experimental thicknesses of samples D and E. As shown in Figure S13b, this formation mechanism explains well the evolution of the layer thicknesses up to 2–2.5 h of the process. From that time on, the estimated thicknesses deviate to higher values. Therefore, a phenomenon that has not significantly influenced the pyrite formation process until now must have become important, reducing the value of the diffusion coefficient. We hypothesize that this change is related to the recrystallization process of the film (i.e., particularly of the pyrite layer).

**Figure 7:**
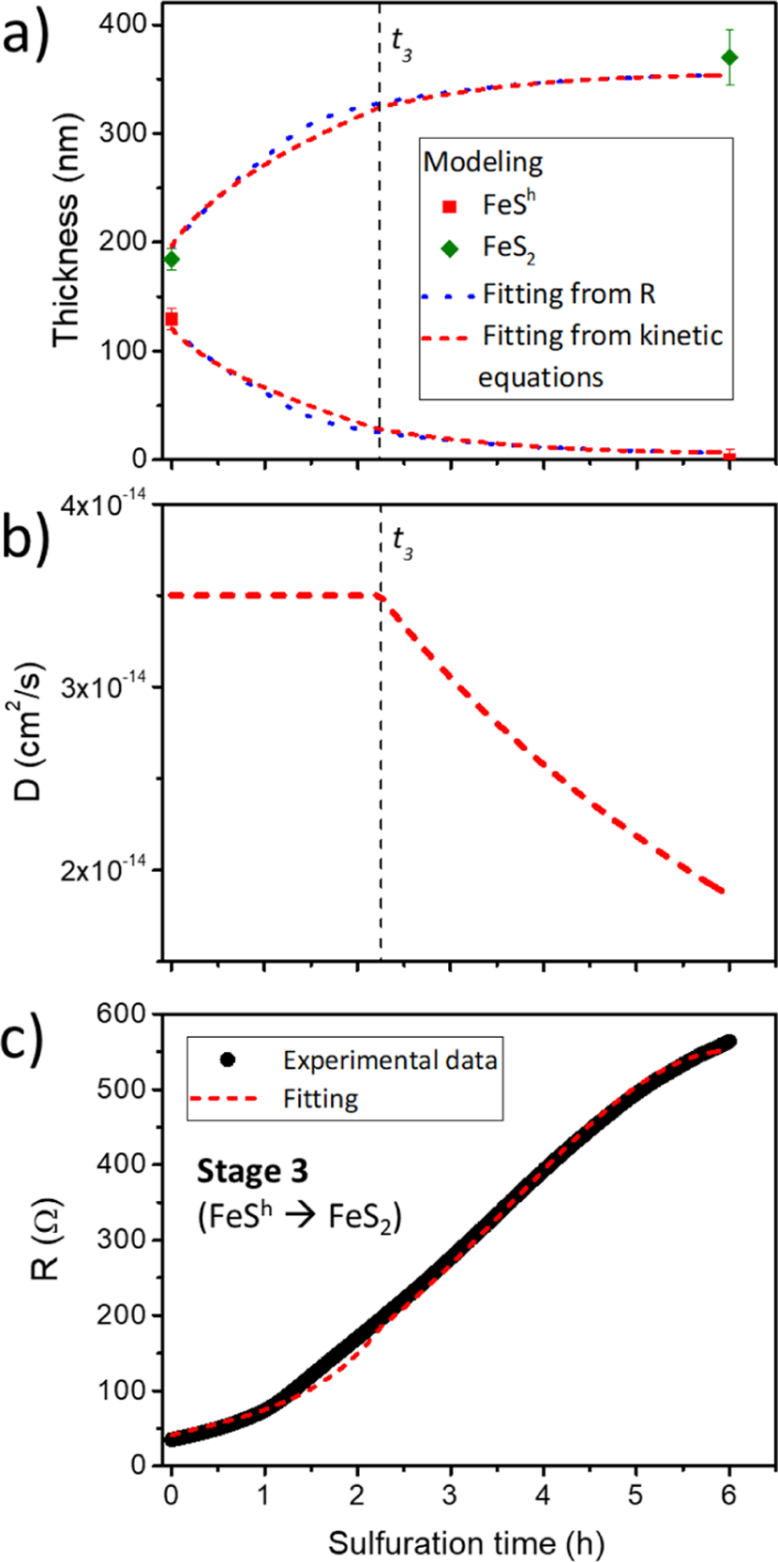
(a) Evolution of the pyrrhotite (Fe_1–*x*_S^h^) and pyrite (FeS_2_) layer thicknesses with the sulfuration time as extracted from the fitting obtained by using the kinetic equations (eqs 17 and 18, in red) and the resistance evolution shown in (c) (eq 20, in blue). (b) Diffusion coefficient as a function of the sulfuration time. The black, vertical dashed lines in (a,b) indicate the time (*t*_3_) when the change in the diffusion coefficient regime occurs. (c) Electrical resistance of the sample. The black circles represent the experimental data obtained from sample E during the sulfuration, while the red dashed lines show the fitted values obtained according to expressions19.

As previously discussed, the recrystallization of pyrite thin films strongly depends on *T*_s_ and *t*_s_.^22,28,29,31,32,43,58^ However, since *T*_s_ remains at its stabilized maximum value during stages 1 and 2 and both present notably shorter duration (∼1.7 and ∼0.4 h respectively) than stage 3 (∼6 h), the annealing time must be the most influential parameter in the recrystallization. This process would induce some changes in the diffusion mechanism in two ways. On the one hand, by reducing the effective diffusion through grain boundaries as the pyrite crystallite size increases^22,27,32^ (which could also be related to a shift in the diffusion mechanism from grain boundaries to grain bulk). And, on the other hand, by sulfur diffusion toward a thinner, less ordered pyrrhotite. The latter factor would be related to the low diffusion coefficient estimated from the discussion of interstitial sulfur toward the pyrrhotite thin film layer, particularly when compared to annealed crystals.^57^ We only have experimental evidence of the first mechanism, as we and other research groups have repeatedly reproduced it at the same temperature as the one used in this work. In order to tentatively estimate the influence of the recrystallization process on the pyrite formation mechanism, we consider that a certain volume of the pyrite thin film is formed by recrystallized grains (*V*_G_) and that the rest has no crystallized material (*V*_V_), including here the gran boundiries region where the diffusion will take place. In this way, the total volume of the sample will be the sum of *V*_G_ and *V*_V_. Then, by applying Avrami’s law to the growth of the volume occupied by grains and expressing the result in terms of not yet recrystallized material (i.e., gran bounduries, *V*_V_),^58^ we obtain
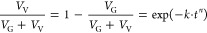
15where *n* is the Avrami coefficient and *k* is a constant related to the nucleation rate and growth of the volume occupied by grains. It is important to note that the Avrami coefficient here refers to a reaction time where the FeS_2_ layer is continuously growing, which potentially can lower this coefficient. However, the pyrite growth rate continuously reduces during this step, being much lower than at the beginning of stage 3. Thus, considering that the diffusion coefficient is proportional to the volume of grain boundaries (i.e., no crystallized material *V*_V_), we can write
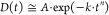
16where *A* is a constant. In this way, the sulfuration process during stage 3 will initially be controlled by a Wagner-type diffusion process with a constant diffusion coefficient, *D*_o_. However, from *t*_3_ on, it is controlled by a time-dependent diffusion coefficient
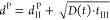
17
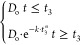
18where *D*_o_ = 3.5 × 10^–14^ cm^2^/s, *d*_II_^P^ is 184 nm, corresponding to the pyrite thickness when starting stage 3 (sample D). And *t*_3_ = 2.3 h, the time at which the diffusion with constant coefficient starts to fail (see Figure S13b). The goodness of the thicknesses fit using these parameters is shown in Figure 7a. Figure 7b plots the time evolution of the diffusion coefficient during the recrystallization process until all hexagonal pyrrhotite is consumed in stage 3.

#### 3.3.2 Modeling of the Reaction: R as a Function of the Sulfuration Time and Comparison with Experimental Results

Similar to what we have done in stage 2, we can fit the electrical resistance of the film by using the time evolution of Fe_1–*x*_S^H^ and FeS_2_ thicknesses. This results in the following expression
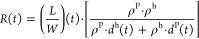
19where the time evolution of  geometric factor during stage 3 is unknown. It could be fitted by different curves (e.g., exponential, linear, or sigmoid) as only two experimental points are known (samples D and E). The final result, however, does not depend on the chosen curve for the geometric factor, as shown in Figure S11 in the Supporting Information In this way, Figure 7c shows a good agreement between the experimental data and the calculated values of *R* (eq 19), validating the assumptions made in our kinetic model.

Furthermore, we highlight that the inverse strategy, i.e., to calculate the thickness evolution from the operando experimental data, is also valid and yields equivalent results. Thus, eq 19 can be modified to obtain the evolution of pyrite film thickness (and indirectly, the pyrrhotite thickness)
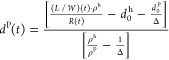
20

The results are depicted in Figure 7a, where the fit of Fe_1–*x*_S^H^ and FeS_2_ layer thicknesses can be directly compared to that using the kinetic model, showing an almost perfect correspondence. Moreover, the Seebeck coefficient increases until its constant value of ∼90 μV/K during this stage is reached. The Seebeck coefficient during this stage could not be measured for this specific sample. However, its calculated time evolution fairly corresponds to the expected one when compared to other samples sulfurated under virtually equivalent conditions (see Figure S14).

### 3.4 Defects in Pyrite Thin Films

The proposed kinetic model and reaction mechanism for the complete sulfuration of metallic Fe into FeS_2_ thin films involves two main steps that are characterized by diffusion of the Fe vacancies (Fe → Fe_1–*x*_S)^38^ and diffusion of interstitial sulfur (Fe_1–*x*_S → FeS_2_). In both cases, the reaction mechanisms are associated with the creation/annihilation of positive charge carriers (i.e., holes). Moreover, the transport properties of undoped pyrite thin films are well-known to be related to intrinsic lattice defects. Whereas iron vacancies (V_Fe_) act as acceptors and generate p-type carriers, the donor S vacancies (V_S_) contribute to negative n-type carriers. Both defects will likely develop under the drawn model, as the conversion of all the species is never complete in each elementary reaction. Furthermore, many bulk characterization techniques used to know the film composition provide average S/Fe ratios (e.g., SEM–EDX^14,27,32^ or XPS^28^ with standard X-ray sources). In other words, they cannot discern between iron defective film caused by V_Fe_ or an excess of interstitial sulfur (S) accumulated at the film grain boundaries, requiring local probes to confirm the latest case.^21,22^ Besides, the signs of the Seebeck and Hall coefficients are not directly conclusive in determining the p or n conductivity type of pyrite thin films. In fact, they show all possible combinations for films grown under slightly different experimental conditions. Two possible explanations have been proposed to try to understand this issue. On the one hand, Zhang et al.^24^ have proposed that the low electron mobility of pyrite thin films compared to that in n-type single crystals (both with V_Fe_ as the main lattice defect) induces a change from diffusive to hopping electronic transport, which results in an apparent p-type crossover.^24,25^ On the other hand, Ares et al.^22,58^ proposed a two-band conduction mechanism operating in the films, with donor (V_S_) and acceptor (V_Fe_) states, that could explain the switch in the Seebeck and Hall coefficients signs.

In order to gather more information about the nature of these intrinsic defects, same samples were sulfurated for 20 more hours after stage 3 (see Figure S2 and ref (27)) under the same experimental conditions. This step implies continuing the pyrite recrystallization process (we will call this step stage 4). The Seebeck coefficient increases from ∼65 to ∼100 μV/K, while *R* increases by a factor of 1.6. By applying the two-band model proposed by Ares et al.^22^ to the Seebeck results, the n/p ratio between the densities of n and p charge carriers decreases by about 9%. This implies that the number of n-type carriers decreases via the formation of V_Fe_ or annihilation of V_S_. However, the formation of V_Fe_ will unlikely occur, as they are created during stage 1. In contrast, sulfur atoms in interstitial positions would likely pick up an electron from the conduction band, ionizing and occupying a lattice position
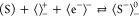
IV

As a result of the above reaction, an V_S_ has been annihilated and an electron has been removed from the conduction band. EDX measurements of samples quenched at the beginning and the end of stage 4 show a corresponding variation of about 10% in the S/Fe ratio,^27^ thus supporting this mechanism. We note that the consumption of an interstitial sulfur atom should not affect the pyrite structure. A new interstitial sulfur atom will be created to maintain the equilibrium with the external S_2_ gas as long as the experimental conditions remain unchanged. Nevertheless, this simplified model is not able to explain the changes in *R* just as a function of the n/p (S/Fe) ratio. Therefore, an additional phenomenon that promotes a decrease in the density of both n and p charge carriers in a very similar proportion must exist. This way, the resistivity would increase without changing the Seebeck coefficient or the S/Fe ratio. This phenomenon could be related to carrier recombination between bands, forbidden band levels, surface traps, grain boundaries, or other types of lattice defects.

**Figure 8:**
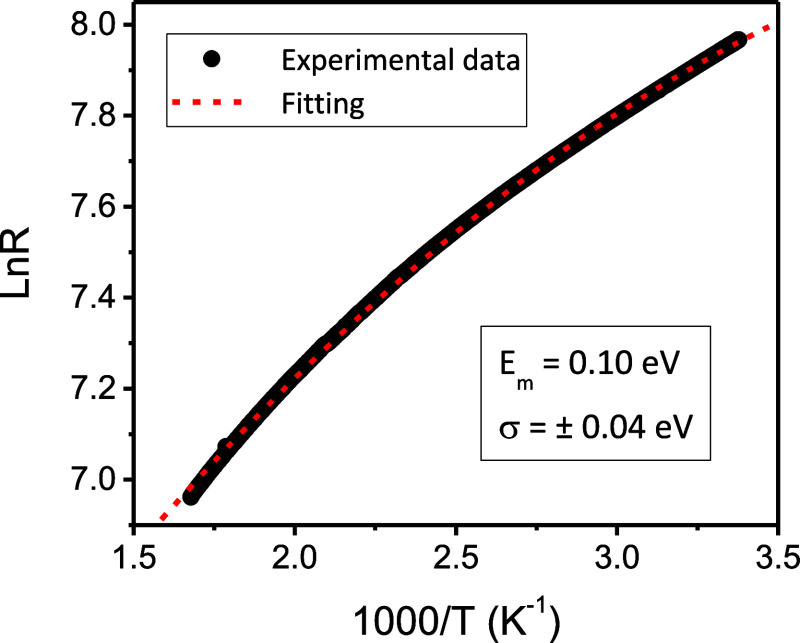
Arrhenius-type presentation of the electrical resistance (*R*) experimental data obtained during pyrite recrystallization after completing 20 h of recrystallization (stage 4). The dashed line indicates the fit obtained by taking the natural logarithm of expression21. The inner inset shows the *E*_m_ and σ obtained values.

Figure 8 presents the Arrhenius-type plot of *R* as a function of temperature during the sample cooling process once stage 4 is completed. Ares et al.^59^ previously discussed the curvature of similar plots, arguing that the energy levels created by iron vacancies (V_Fe_ > V_S_) in the forbidden band transform it into a narrower band.^59,60^ This new energy must be characterized by an average energy value, *E*_m_, as well as by a dispersion of the energy of this level, σ. Therefore, *R* can be expressed as
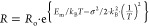
21

By applying natural logarithm and fitting the curve in Figure 8 we obtain *E*_m_ = 0.10 eV and σ = ±0.04 eV. These values are similar to those previously reported elsewhere^59^ for pyrite thin films sulfurated under different experimental conditions than those used here. The relatively large value of σ reflects a substantial dispersion of energies in the acceptor band. This fact seems to agree with the existence of a high interaction between cation vacancies and the fact that these can be single or double-ionized. Moreover, if the same analysis is performed on sample E (i.e., end of stage 3, thus without the subsequent annihilation of V_S_ and complete recrystallization), we obtain the same *E*_m_ and σ values. As for the totally recrystallized FeS_2_ film, this fact indicates that (i) the density of V_Fe_ must be higher than V_S_, (ii) the resistance (*R*) evolution with temperature is governed by the thermal excitation-desexcitation of electrons from the valence band to the acceptor band, and (iii) grain boundaries, i.e., accumulated interstitial sulfur atoms, do not play an important role in the conductivity of pyrite thin films.

We note that our experimental setup does not allow in situ measurements of the Hall coefficient or ex situ low-temperature characterization as in.^24,25^ Therefore, complementary measurements that would help to determine the electron transport mechanism unambiguously are lacking.

## 4 Conclusions

We have shown that the sulfuration of pyrrhotite to transform it into pyrite takes place in two distinct stages: in the first one, the conversion of orthorhombic pyrrhotite to pyrite takes place while the hexagonal pyrrhotite phase remains unaltered (Fe_1–*x*_S^O^ → FeS_2_). In the second one, the final transformation of hexagonal pyrrhotite to pyrite (Fe_1–*x*_S^H^ → FeS_2_) occurs. In both cases, the pyrrhotite sulfurates into pyrite at the internal Fe_1–*x*_S/FeS_2_ interface via interstitial sulfur diffusion through the pyrrhotite layer. Consequently, the pyrite layer grows from bottom to top. The reaction mechanism at each stage has been validated by using the corresponding kinetic model to fit the experimental data on time evolution of the film thickness and transport properties (*R* and *S*_th_). In this way, during stage 2 (Fe_1–*x*_S^O^ → FeS_2_), the adsorption of S_2_ initially limits the reaction at the sample surface, subsequently changing to a Wagner-type diffusion process independent of the S_2_ partial pressure. The same mechanism is valid for stage 3, even though the sulfur diffusion coefficient gradually decreases at the moment that the recrystallization of the pyrite film starts to be significant.

This description complements the previous work^37^ focused on the initial sulfuration of metallic iron into hexagonal and orthorhombic pyrrhotite phases (Fe → Fe_1–*x*_S^H^ + Fe_1–*x*_S^O^); thus, the detailed description of the reaction mechanism that governs the sulfuration of iron into pyrite is completed. We note that the appearance and detection of intermediate pyrrhotite phases correspond to particular experimental conditions for which the sulfuration reaction has been slowed down. Importantly, these intermediate species can be hindered by using other experimental conditions, mainly higher S_2_ partial pressures or substrate temperature, which in turn will determine the final characteristics of the sulfurated films. Therefore, further experimental work should be dedicated to systematically exploring the effects of sulfuration conditions and how they affect the duration and development of the different reaction stages described in this series of articles and, thus, the presence and role of defects in pyrite films. All in all, the proposed global reaction mechanism can explain the microstructure of pyrite films (i.e., Kirkendall effect and formation of a porous layer), the presence of intrinsic defects, such as iron and sulfur vacancies, and the accumulation of interstitial sulfur at the grain boundaries. In this regard, the conductivity of pyrite films is tentatively explained by employing a two-band model. In this model, the Seebeck coefficient behavior and S/Fe ratio evolution can be successfully explained during the pyrite recrystallization stage. In order to understand the influence of the experimental conditions on the transport properties of pyrite thin films, more in situ experiments should be done to clarify how the creation of intrinsic lattice defects is modified.

## Supporting Information Available

Partial pressure of S_2_ molecular species. Fe → FeS → FeS_2_ transformation and XRD patterns of B, C, D, and E samples Pyrrhotites: crystallographic details. Besson’s notation TEM–EDX cross-section images of sulfurated film at equivalent conditions to those of sample D Kinetic equations Determination of the independent fitting parameters for stages 2 (Fe_1–*x*_S^O^ → FeS_2_) and 3 (Fe_1–*x*_S^H^ → FeS_2_) of the sulfuration process Alternative modeling by considering diffusion as the limiting process during stage 2 (Wagner diffusion) Alternative modeling by considering Wagner diffusion with constant diffusion coefficient as the limiting process during stage 3 (PDF)
