# Supplementary material for: Reaction Mechanism and Kinetic Model of the Transformation of Iron Monosulfide Thin Films into Pyrite Films
Source: J Phys Chem C Nanomater Interfaces. 2025 Feb 19;129(9):4724–37. doi: 10.1021/acs.jpcc.4c08227 (PMC11892422; doi:10.1021/acs.jpcc.4c08227)
Supplement: Supplementary file 1 — jp4c08227_si_001.pdf [file jp4c08227_si_001.pdf]

Supporting Information

Reaction Mechanism and Kinetic Model of the Transformation of Iron Monosulfide Thin Films into Pyrite Films

Carlos Morales[1], Antonio Pascual[2], Dietmar Leinen[3], Gabriel Luna-López[2], Jose R. Ares[2], Jan Ingo Flege[1], Leonardo Soriano[4,5], Isabel J. Ferrer[2,5] and Carlos Sanchez[2,5].*

[1] *Applied Physics and Semiconductor Spectroscopy, Brandenburg University of Technology Cottbus–Senftenberg, Konrad-Zuse-Strasse 1, D-03046 Cottbus, Germany*

[2] *Dpto. de Física de Materiales, Facultad de Ciencias, Universidad Autónoma de Madrid, Francisco Tomás y Valiente 7, E-28049 Madrid, Spain.*

[3] *Departamento de Física Aplicada I, Facultad de Ciencias, Universidad de Málaga, Campus Teatinos, Málaga, C.P. 29071, Spain*

[4] *Dpto. de Física Aplicada, Facultad de Ciencias, Universidad Autónoma de Madrid, Francisco Tomás y Valiente 7, E-28049 Madrid, Spain.*

[5] *Instituto Nicolás Cabrera, Universidad Autónoma de Madrid, Francisco Tomás y Valiente 7, E-28049 Madrid, Spain*

[*] *Corresponding author: email: carlos.moralessanchez@b-tu-de*

The partial pressure of S_2 molecular species.

In our experimental setup, the S_2 partial pressure (P_{S2}) will depend: (1) on the total pressure, P_T, which evolution with temperature is well-known[1,2] and corresponds to that of the first furnace (sulfur basket temperature, $T_{sulfur\ basket}$); and (2) on the temperature (T_{Sample}) of the second furnace, where the sulfuration process takes place under an unsaturated atmosphere enriched in S_2 species[3]. In this way, P_{S2} could be expressed as $P_{S2} = f(P_T,T_S)$. Considering the separated P_{S_2} = P_T curves from H. L. Barnes et al.[3] as a function of T (P_T, total sulfur pressure, is constant in each case), we have fitted the P_{S2} curves by a Boltzmann expression dependent only on T (where T is the sulfuration temperature, T_S in our particular experimental setup).

$$P_{S_2} = \frac{A_1 - A_2}{1 + e^{(T-x_0)/c}} + A_2 \qquad \text{(S1)}$$

The parameters A_1 and A_2 represent the minimum and maximum S_2 partial pressures, respectively. Since, in all cases, the atmosphere composition consists of the molecular species S_2 at sufficiently high temperatures, the parameter A_2 corresponds to the total pressure P_T of each specific curve. Moreover, the parameter A_1 indicates by definition the initial value (at low T) of P_{S_2}, but it cannot be accurately defined since the experimental data do not record it[3]. Thus, these values must be taken just as mathematical values. The calculated x_0, c, and A_1 are shown in the following table:

P_T (atm)	x_0 (K)	c (K)	A_1 (atm)
1	987.63	64.91	$2.82\cdot10^{-2}$
10^{-1}	872.24	45.07	$1.53\cdot10^{-3}$
10^{-2}	787.31	44.28	$6.23\cdot10^{-5}$
10^{-3}	695.50	35.01	$2.64\cdot10^{-6}$
10^{-4}	651.31	34.23	$1.43\cdot10^{-7}$
10^{-5}	590.67	24.31	$1.12\cdot10^{-7}$
10^{-6}	527.38	19.24	$4.97\cdot10^{-9}$
10^{-7}	513.59	24.40	$2.60\cdot10^{-10}$
10^{-8}	470.31	17.61	$9.46\cdot10^{-11}$
10^{-9}	434.04	17.34	$1.03\cdot10^{-11}$

Table S1. Fitting values for x_o, c and A_1 by considering data from H. L. Barnes et al.[3] and expression S1.

Subsequently, from Table S1, general expressions for x_o, c, and A_1 can be deduced as a function of P_T:

$$x_0 = 976.421 + 102.523 \cdot log(P_T) + 4.840 \cdot (log(P_T))^2 \text{(S2)}$$

$$c = 60.797 + 9.934 \cdot log(P_T) + 0.581 \cdot (log(P_T))^2 \qquad \text{(S3)}$$

$$log(A_1) = -1.936 + 1.038 \cdot log(P_T) \qquad \text{(S4)}$$

As the evolution of P_T with temperature (in our particular experimental case, sulfur basket temperature) is well-known[1,2], P_{S_2} can be expressed by only two experimental parameters. These parameters are: the temperature at the first oven (sulfur basket) and temperature at the second oven (sample temperature, sulfuration temperature).

Thus, obtaining:

$$P_{S_2} = \frac{A_1 - P_T}{1 + e^{(T_S - x_0)/c}} + P_T \qquad \text{(S5)}$$

Figure S1 shows a three-dimensional plot of P_{S_2} as a function of total pressure (P_T) and temperature.

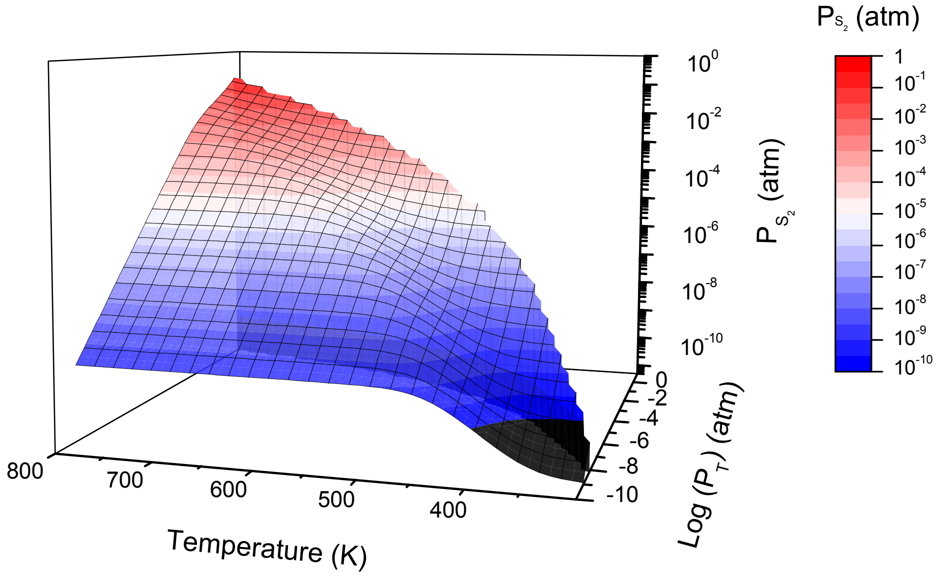

Figure S1. Three-dimensional plot of the molecular S_2 partial pressure (P_{S_2}) as a function of temperature (T, in our case, sample temperature T_s) and total sulfur pressure (P_T) in the system.

The Fe → FeS → FeS_2 transformation and XRD patterns of B, C, D, and E samples

The qualitative description of the Fe→FeS and FeS→FeS_2 transformations in terms of phases evolution and changes on the Seebeck coefficient can be found in Pascual et al. work[4]. In Figure S2, we show the four representative samples used in the present work to explain the reaction and kinetic model of the FeS→FeS_2 transformation by sulfuration, divided into stage 2 ($Fe_{1-x}S^O$→FeS_2) and stage 3 ($Fe_{1-x}S^H$→FeS_2). The XRD patterns are reproduced from ref. 4. Note that the Seebeck coefficient (S_{th}) full curve in Figure S2a corresponds to a completely sulfurated sample. Reaction stages are only tentatively indicated. Stage 1 (Fe→$Fe_{1-x}S$) has been extensively discussed elsewhere[5].

In Figure S2, the starting time (t=0) is set when the ovens start their heating ramp. However, we highlight that in this work figures, t=0 is set at the moment when each stage starts (as indicated in the text).

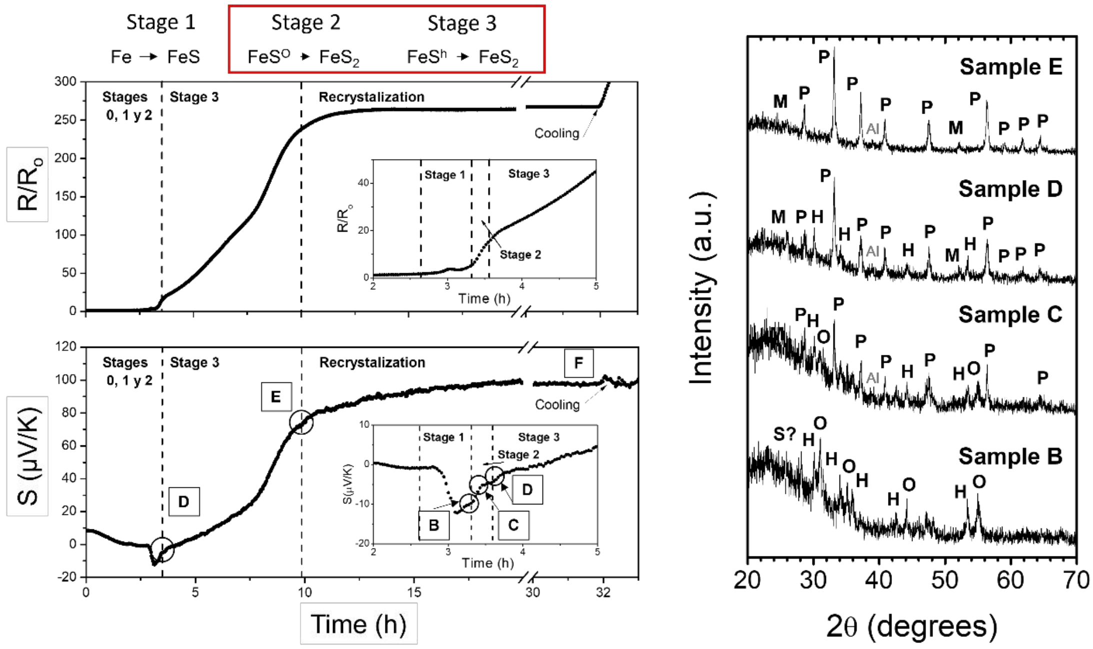

Figure S2. Normalized electrical resistance, R/R_0, and Seebeck coefficient (S_{th}) of the film as a function of its sulfuration time. The labels B, C, D, and E indicate the final moment of the respective sulfuration stages. The Fe film annealing corresponds to the time < 2.5 h. On the right hand, grazing angle XRD diffractograms of the same samples. Fe: iron (JCPDS 06–0696); P: pyrite (JCPDS 42–1340); M: marcasite (JCPDS 02–0908); H: hexagonal pyrrhotite; O: orthorhombic pyrrhotite (see crystallographic details in Table S2 and Figure S4); Al: aluminum from the sample holder of the XRD diffractometer. Adapted with permission from J. Phys. Chem. C 2014, 118, 46, 26440–26446. Copyright 2014 American Chemical Society.

Pyrrhotites: crystallographic details.

	Hexagonal pyrrhotite	Orthorhombic pyrrhotite
**Crystallographic phase**	(190) P$\bar{6}$2c	(62) Pnma
**Lattice constants**	a = 5.9409 Å<br>b = 5.9409 Å<br>c = 11.3600 Å	a = 5.6500 Å<br>b = 3.3130 Å<br>c = 5.7380 Å
**Lattice angles**	α = 90°<br>β = 90°<br>γ = 90°	α = 90°<br>β = 90°<br>γ = 90°
**Wyckoff positions**	**Fe** X = 0.3781<br>Y = 0.0543<br>Z = 0.1231	**Fe** X = 0.0130<br>Y = 1/4<br>Z = 0.2009
	**S** X = 0<br>Y = 0<br>Z = 0	**S** X = 0.2190<br>Y = 1/4<br>Z = 0.5792

Table S2. Crystallographic details of hexagonal[6] and orthorhombic[7] pyrrhotites.

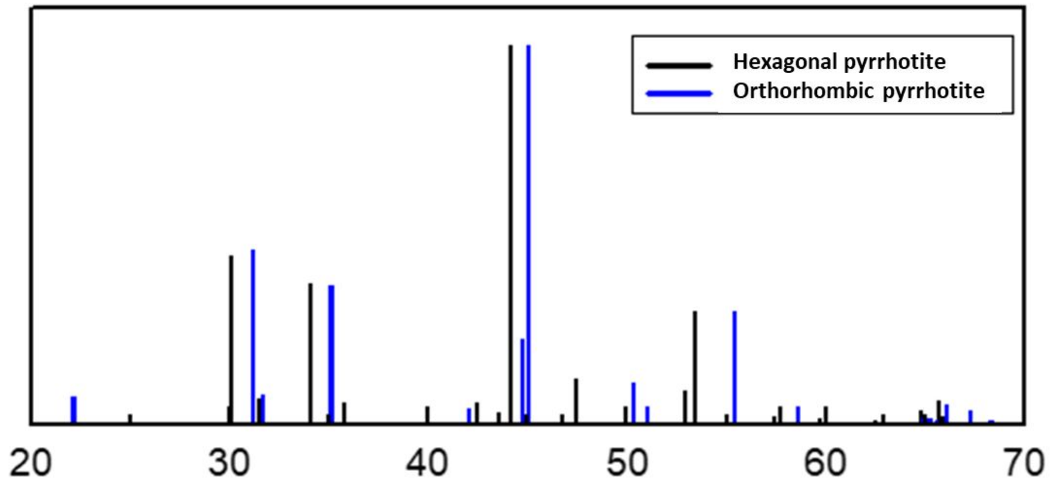

Figure S3. Calculated diffraction patterns[6,7] from the crystallographic data presented in Table S2.

Besson's notation

$\langle Fe^{2+}\rangle_{2+}^{0}$: Fe^{2+} ion occupying a cationic site

$\langle S^{2-}\rangle_{2-}^{0}$: S^{2-} ion occupying an anionic site

$\langle S_2^{2-}\rangle_{2-}^{0}$: S_2^{2-} ion occupying an anionic site

$\langle\ \rangle_{2+}^{2-}$: cationic vacancy

$\langle\ \rangle_{2-}^{2+}$: anionic vacancy

$(Fe^{2+})^{2+}$: Fe^{2+} ion occupying an interstitial site

(S): S neutral atom occupying an interstitial site

$(S^{-})^{-}$: S^{-} ion occupying an interstitial site

$(e^{-})^{-}$: interstitial electron

$\langle e^{-}\rangle_{2-}^{+}$: electron occupying an anionic position

$(h^{+})^{+}$: interstitial hole

$\langle h^{+}\rangle_{2+}^{-}$: hole occupying an anionic position

$\langle\ \rangle$: neutral iron vacancy within the metallic iron matrix

Real charge: the charge that the ion, electron, hole, etc., has. It is represented as a superscript next to the symbol (Fe^{2+}, h^{+}, e^{-}, etc.).

Net site charge: the charge associated with a specific net site. It is represented by a subscript next to the network site symbol. The interstitial position has a charge equal to zero and is not indicated. For example, a lattice position of an Fe cation will have a 2+ charge associated with it, and will be labelled as $\langle\rangle_{2+}$.

Effective charge: the difference between real charge and net site charge, i.e. the charge of that specific identity as seen by the crystal. For example, a hole with real charge 1+ in a cationic position with net site charge 2+, will present an effective charge of (1+)-(2+)= -1. The effective charge is represented as a superscript of the lattice site (following the example above: $\langle h^{+}\rangle_{2+}^{-}$).

TEM-EDX cross-section images of sulfurated film at equivalent conditions to those of sample D

Figure S4 shows the false color EDX-TEM cross-section images performed on a sulfurated layer at conditions equivalent to sample D in the manuscript (see Figure S2). The EDX-TEM composition mappings correspond to those shown in Figures 1b and 1e in the manuscript.

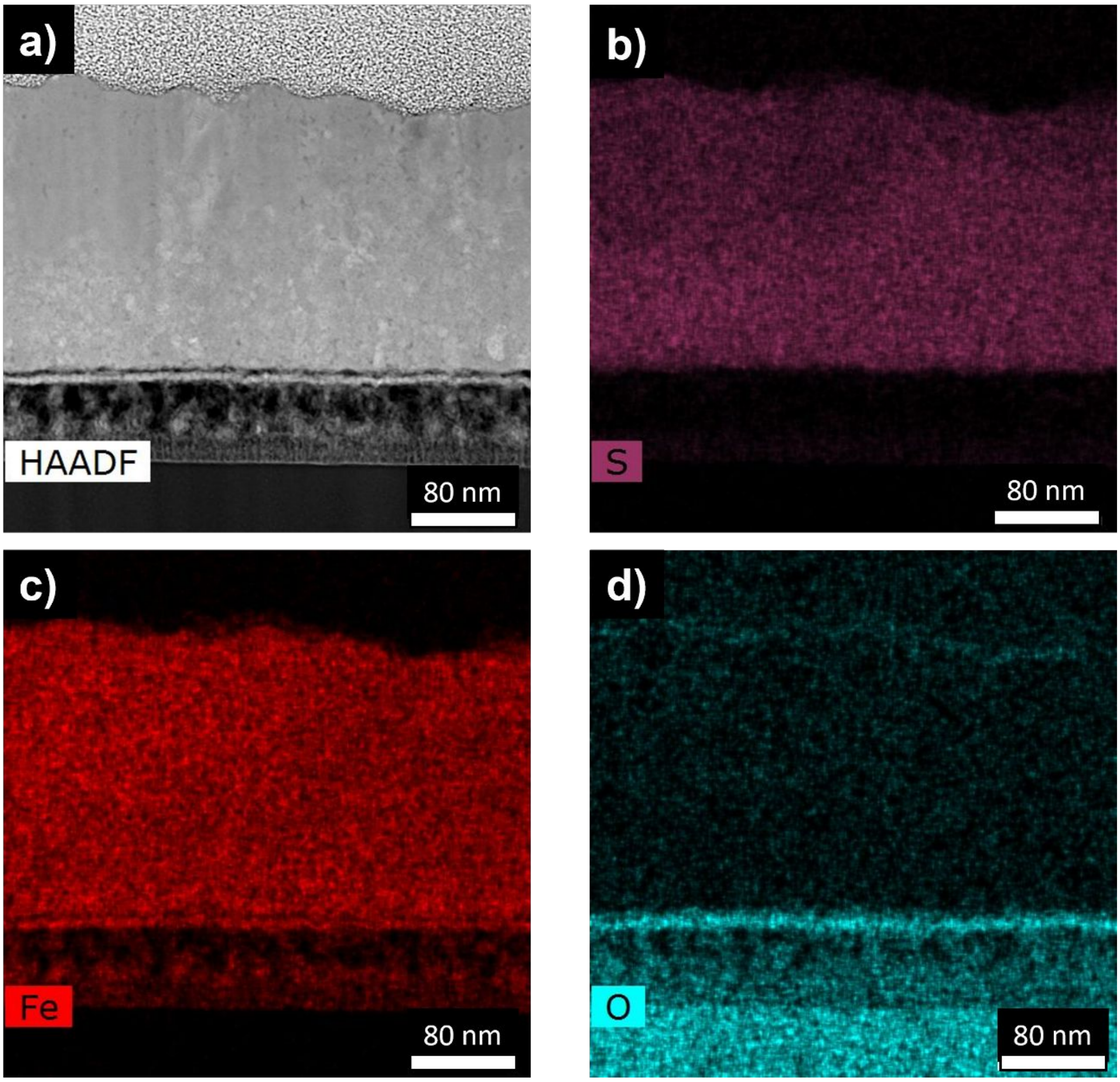

Figure S4. a) Cross-section TEM-HAADF image of sulfurated thin films at equivalent conditions to those applied to sample D (see Figure S2), equal to Figure 1b in the manuscript. Panels b), c), and d) show the corresponding compositional EDX mappings of sulfur (violet), iron (red), and oxygen (turquoise).

Kinetic equations

(I) S_2 adsorption on pyrrhotite surface

A_0: maximum number of free surface sites for sulfur adsorption (maximum number of surface Fe atoms).

A(t): number of available sites at time t for sulfur adsorption (number of pyrrhotite free $\langle Fe^{2+}\rangle^0_{2+s}$ cations at the surface).

A_0-A(t): number of occupied sites (adsorbed gas atoms or occupied Fe atoms).

Definition of the total fraction of occupied surface active sites (θ) as a function of time:

$$\theta(t) \equiv \frac{A_0 - A(t)}{A_0} = 1 - \frac{A(t)}{A_0} \qquad \text{(S6)}$$

The kinetic equation for the direct and inverse reactions are:

$$\vec{v}_a = \vec{k}_a \cdot P_{S_2}^{1/2} \cdot (1-\theta) \qquad \text{(S7)}$$

$$\overleftarrow{v}_a = \overleftarrow{k}_a \cdot \theta \qquad \text{(S8)}$$

where P_{S_2} is the S_2 partial pressure and $\vec{k}_a$ and $\overleftarrow{k}_a$ are the kinetic constants of the direct and inverse reactions, respectively. The total velocity is:

$$v_a = \vec{v}_a - \overleftarrow{v}_a = \vec{k}_a \cdot P_{S_2}^{1/2} \cdot (1-\theta) - \overleftarrow{k}_a \cdot \theta \qquad \text{(S8)}$$

Under equilibrium conditions $\vec{v}_a = \overleftarrow{v}_a$. Then, the fraction of occupied surface active sites under equilibrium conditions (θ_∞) may be defined as:

$$\theta_\infty = \frac{k_a \cdot P_{S_2}^{1/2}}{1 + k_a \cdot P_{S_2}^{1/2}} \qquad \text{(S9)}$$

Where $k_a = \frac{\vec{k}_a}{\overleftarrow{k}_a}$, and rewriting equation S8:

$$v_a = \vec{k}_a \cdot P_{S_2}^{1/2} \cdot \left(1 - \frac{\theta}{\theta_\infty}\right) \qquad \text{(S10)}$$

(II) Reaction at the sample external interface (sulfur-pyrrhotite)

(II.a) Charge exchange. As explained in the main text, the final velocity of this step is given by:

$$v_{eI} = \vec{k}_{eI} \cdot \theta - \overleftarrow{k}_{eI} \cdot D_1^3 \qquad \text{(SI 11)}$$

where D_1 is the concentration of cationic vacancies and interstitial holes, and $\vec{k}_{eI}$

and $\overleftarrow{k}_{eI}$ are the kinetic constants of the direct and inverse reactions, respectively.

(II.b) Ionic exchange. As explained in the main text, after defining C_e as the concentration of interstitial sulfur atoms, (S), (where $C_e < D_1$), and $\overrightarrow{k}_{eII}$ and $\overleftarrow{k}_{eII}$ as the kinetic constants of the direct and inverse reactions, the kinetic equation of this cation exchange may be written:

$$v_{eII} = \overrightarrow{k}_{eII} \cdot CD_1^3 - \overleftarrow{k}_{eII} \cdot (1-\theta) \cdot C_e \qquad \text{(SI 12)}$$

In order to combine equations S11 and S12 in a single expression for the whole step II, we assume that the cation vacancies do not participate directly in the reaction, and thus stationary conditions can be applied $\left(\frac{dD_1}{dt} = 0\right)$. Therefore:

$$C_1^3 = \frac{\overrightarrow{k}_{eI} \cdot \theta + \overleftarrow{k}_{eII} \cdot (1-\theta) \cdot C_e}{\overrightarrow{k}_{eII} + \overleftarrow{k}_{eI}} \qquad \text{(SI 13)}$$

Now S12 can be written as:

$$v_e = k_e \cdot \theta - k'_e \cdot (1-\theta) \cdot C_e \qquad \text{(SI 14)}$$

where $k_e \equiv \frac{\overrightarrow{k}_{eI} \cdot \overrightarrow{k}_{eII}}{\overrightarrow{k}_{eII} + \overleftarrow{k}_{eI}}$ and $k'_e \equiv \overrightarrow{k}_{eII} - \frac{\overrightarrow{k}_{eII} \cdot \overleftarrow{k}_{eII}}{\overrightarrow{k}_{eII} + \overleftarrow{k}_{eI}}$. As for step I, C_e can be expressed in terms of C_e^{∞}, i.e., equilibrium conditions where $v_e = 0$:

$$C_e^{\infty} = \frac{k_e \cdot \theta}{k'_e \cdot (1-\theta)} \qquad \text{(SI 15)}$$

Therefore, the final expresión for the velocity is:

$$v_e = k_e \cdot \theta \cdot \left(1 - \frac{C_e}{C_e^{\infty}}\right) \qquad \text{(SI 16)}$$

(III) Diffusion of sulfur atoms through the formed pyrrhotite layer.

The complete information is explained in the main text. The final velocity is given by:

$$J_y = -D\frac{\partial C}{\partial d_P} \cong -D\frac{(C_i - C_e)}{d_P} = -D\frac{(C_i - C_e)}{d_{Po} - \frac{d^P}{\Delta}} \qquad \text{(S17)}$$

where D is the vacancy diffusion coefficient, C_e and C_i are the sulfur atoms (S) concentration at the external and internal interfaces, respectively, and d_p is the pyrrhotite thickness that the (S) vacancies must go through. Considering that the initial pyrrhotite thickness is $d_{po}$and it is uniquely sulfurating into pyrite, with thickness d^p, the diffusion can be also expressed as a function of pyrite layer thickness by knowing the relationship between pyrrhotite and pyrite molar volumes, $\Delta d^p = \Delta(d_{P_o} - d_p)$

(IV) Reaction at the film internal interface (pyrrhotite-pyrite).

(IV.a) Charge exchange. As explained in the main text, the velocity of this partial reaction is given by:

$$v_{iI} = \vec{k}_{iI} \cdot C_i \cdot E_t^2 - \overleftarrow{k}_{iI} \cdot E_1^2 \qquad \text{(S18)}$$

where $\vec{k}_{iI}$ and $\overleftarrow{k}_{iI}$ are the kinetic constants of the direct and inverse reactions, respectively, and C_i is the interstitial sulfur atoms (S) concentration at the internal interface of the film. Regarding the metallic iron active sites at the internal interface, it is helpful to define:

E_0: maximum concentration of FeS molecules at the internal interface (able to react) of the pyrrhotite layer.

E_m: concentration of FeS molecules in the pyrrhotite layer at the internal interface at time t (maximum number, E_0, minus those that have already undergone charge exchange).

E_1: concentration of anionic vacancies $\langle\ \rangle_-^+$ and interstitial anions $(S^-)^-$ formed after charge exchange at the internal interface.

(IV.b) Ion exchange. As exposed in the main text, the velocity of this partial reaction is given by:

$$v_{iII} = \vec{k}_{iII} \cdot E_1^2 - \overleftarrow{k}_{iII} \cdot (E_0 - E_m) \qquad \text{(S19)}$$

where $\vec{k}_{iII}$ and $\overleftarrow{k}_{iII}$ are the kinetic constants of the direct and inverse reactions, respectively.

As justified for the ion exchange step at the interface, we consider that the anionic vacancies do not explicitly participate in the reaction, and thus, we can apply stationary conditions $\left(\frac{dB_1}{dt} = 0\right)$, obtaining:

$$E_1^2 = \frac{\vec{k}_{iI} \cdot E_m \cdot C_i - \overleftarrow{k}_{iII} \cdot (E_0 - E_m)}{\vec{k}_{iII} + \overleftarrow{k}_{iI}} \qquad \text{(S20)}$$

We can now substitute S20 in S19, resulting in:

$$v_i = k_i \cdot E_m \cdot C_i - k_i' \cdot (E_0 - E_m) \qquad \text{(S21)}$$

where $k_i \equiv \frac{\vec{k}_{iII} \cdot \vec{k}_{iI}}{\vec{k}_{iII} + \overleftarrow{k}_{iI}}$ and $k_i' = \overleftarrow{k}_{iII} - \frac{\vec{k}_{iII} \cdot \overleftarrow{k}_{iII}}{\vec{k}_{iII} + \overleftarrow{k}_{iI}}$. As in all previous reactions, we can express S21 in terms of C_i at equilibrium conditions:

$$v_i = k_i \cdot (C_i - C_i^\infty) \qquad \text{(S22)}$$

with:

$$C_i^\infty = \frac{k_i' \cdot (E_0 - E_m)}{k_i \cdot E_m} \qquad \text{(S23)}$$

$$k_i \equiv \frac{\vec{k}_{iII} \cdot \vec{k}_{iI} \cdot E_m}{\vec{k}_{iII} + \overleftarrow{k}_{iI}} \qquad \text{(S24)}$$

Determination of the independent fitting parameters for stages 2 ($Fe_{1-x}S^O \rightarrow FeS_2$) and 3 ($Fe_{1-x}S^H \rightarrow FeS_2$) of the sulfuration process.

Furnaces temperature and partial pressure of S_2 species

Figures S5 and Fig S6 show the temperatures of both sulfur basket and sample furnaces. By applying the method described in the first section of the S.I., the partial pressure of S_2 species has been calculated.

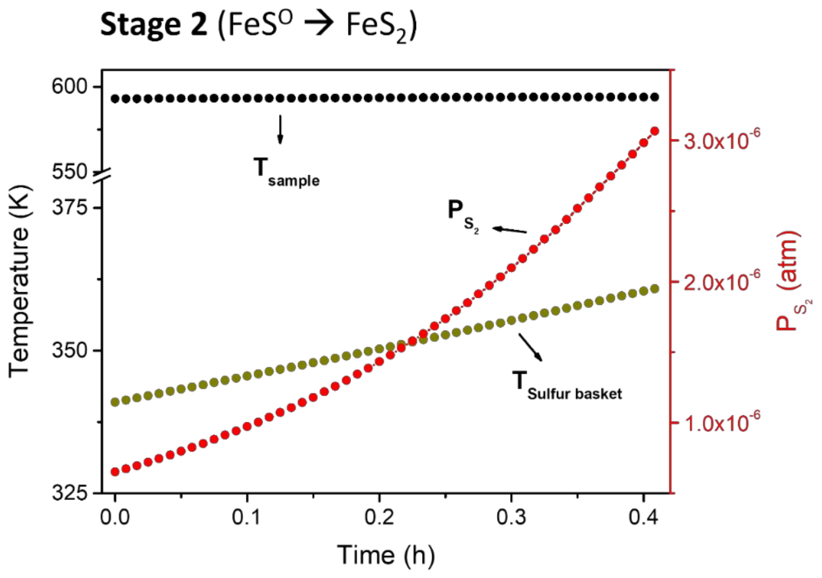

Figure S5. Stage 2 ($Fe_{1-x}S^O \rightarrow FeS_2$): time evolution of the sample and sulfur basket temperatures (left axis) and partial pressure of S_2 species (right axis) during a sulfuration process.

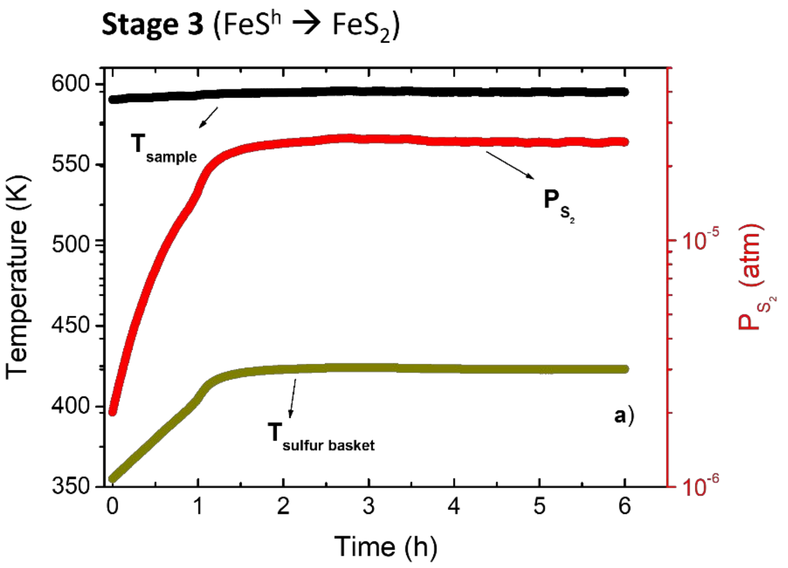

Figure S6. Stage 3 ($Fe_{1-x}S^H \rightarrow FeS_2$): time evolution of the sample and sulfur basket temperatures (left axis) and partial pressure of S_2 species (right axis) during a sulfuration process

Reference samples

Pure hexagonal pyrrhotite sample

Figure S7 shows the XRD diffractogram of a pure hexagonal pyrrhotite sample taken as a reference for the Seebeck's coefficient and resistivity values.

The obtained values are:

$$\rho^{H} = 1.23 \times 10^{-3}\, \Omega \cdot cm \qquad \text{(S25)}$$

$$S^{H} \cong -3\, {}^{\mu V}\!/_{K} \qquad \text{(S26)}$$

However, during the current fitting of stage 2 data, a value for the Seebeck coefficient of -11 μV/K was used, similar to those published elsewhere[8]. This difference could be related to the fact of having less ordered $Fe_{1-x}S^{H}$ during this stage as compared to the high-purity sample shown in Figure S7. This sample was grown under specific conditions to inhibit the formation of orthorhombic pyrrhotite, which enhanced its crystallization. It is worth noting that the fitting of stage 1 data published in our previous work[5] shows equal results for both Seebeck coefficients.

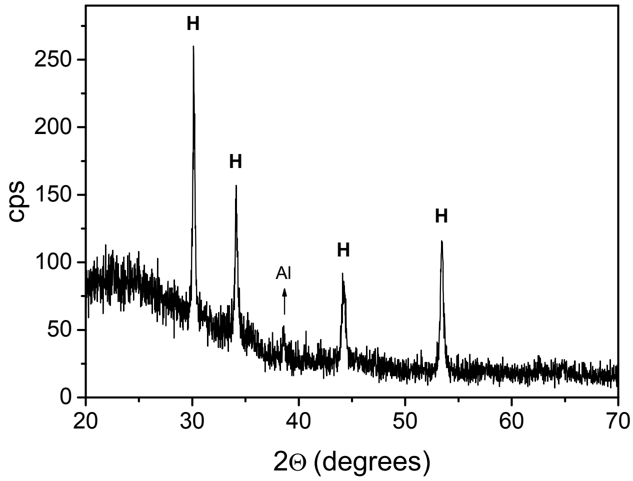

Figure S7. XRD diffractogram of pure hexagonal pyrrhotite sample.

Pure orthorhombic pyrrhotite

Pure orthorhombic pyrrhotite samples could not be obtained. Nevertheless, we prepared films with both hexagonal and orthorhombic phases (without detecting the presence of metallic Fe). We then obtained Seebeck's coefficient and resistivity values of the orthorhombic phase by considering a two parallel layers model:

$$\frac{\rho^{total}}{d^{total}} = \frac{\rho^{O} \cdot \rho^{h}}{\rho^{O} \cdot d^{h} + \rho^{h} \cdot d^{O}} \;\Rightarrow\; \rho^{O} = \frac{\rho^{total} \cdot \rho^{h} \cdot d^{O}}{\rho^{h} \cdot d^{total} - \rho^{total} \cdot d^{h}} \qquad \text{(S27)}$$

$$S^{total} = \frac{\rho^{O} \cdot S^{h} \cdot d^{h} + \rho^{h} \cdot S^{O} \cdot d^{O}}{\rho^{O} \cdot d^{h} + \rho^{h} \cdot d^{O}} \;\Rightarrow\; S^{O} = \frac{\rho^{O} \cdot S^{total} \cdot d^{h} + \rho^{h} \cdot S^{total} \cdot d^{O} - \rho^{O} \cdot S^{h} \cdot d^{h}}{\rho^{h} \cdot d^{O}} \qquad \text{(S28)}$$

,where the thicknesses are obtained by calculating the corresponding phase fraction from DRX diffractograms. We obtain the following values:

$$\rho^O = 7.18 \times 10^{-4}\, \Omega \cdot cm \qquad \text{(S29)}$$

$$S^O \cong -29\, {}^{\mu V}/_{K} \qquad \text{(S30)}$$

Pure pyrite

The obtained experimental values for pyrite at its formation temperature are:

$$\rho^P = 8.86 \times 10^{-2}\, \Omega \cdot cm \qquad \text{(SI 31)}$$

$$S^P \cong 55\, {}^{\mu V}/_{K} \qquad \text{(SI 32)}$$

Electrical model

Figures S8 and S9 show the electrical model applied to interpret the Seebeck's coefficient (S) and electric resistance (R) data as a function of sulfuration time for stages 2 and 3, respectively.

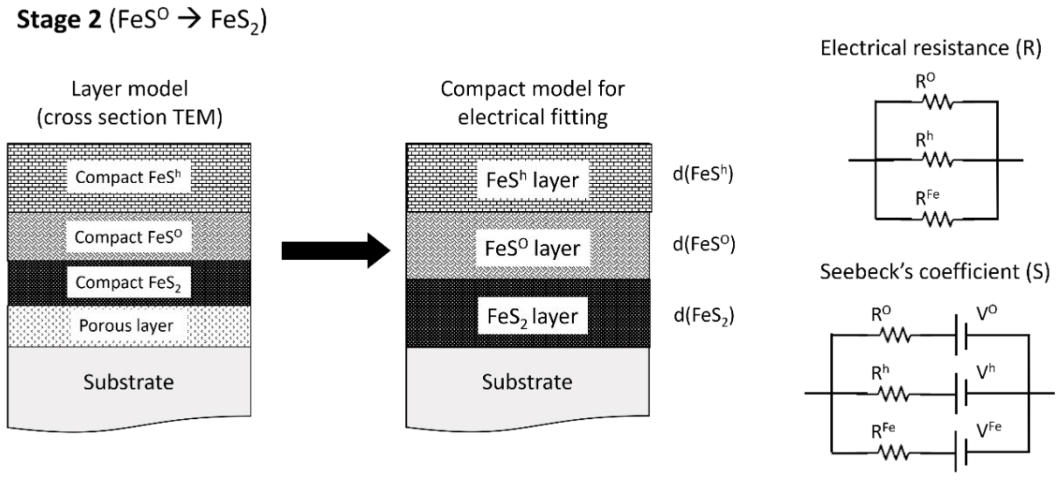

Figure S8. Stage 2 of the sulfuration process. From left to right: cross-section diagram of a general not completely sulfurated sample. A simplified diagram of the "three-parallel layers" model. Finally, the equivalent electrical circuits used to calculate the electrical resistance and Seebeck coefficient of the films according to the three-parallel layer model without porous layer.

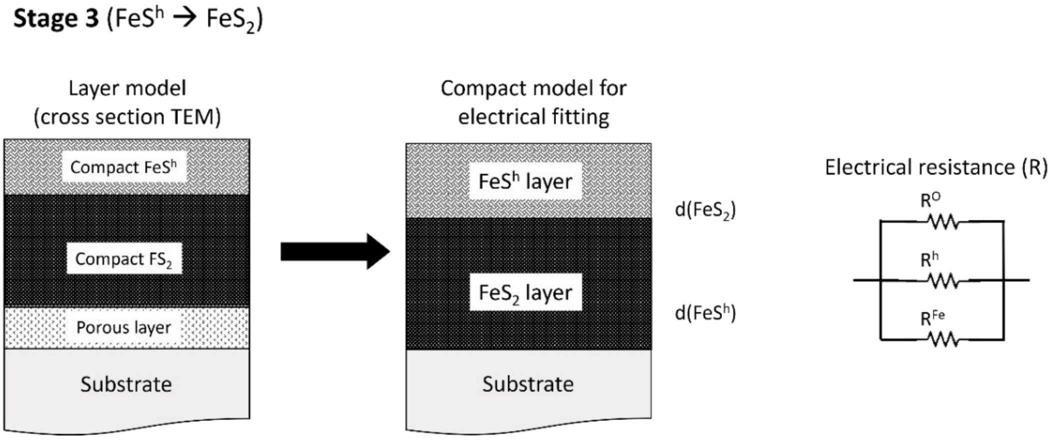

Figure S9. Stage 3 of the sulfuration process. From left to right: cross-section diagram of a general not completely sulfurated sample. A simplified diagram of the "three-parallel layers" model. Finally, the equivalent electrical circuit used to calculate the electrical resistance of the films according to the three-parallel layer model without porous layer.

Geometrical factor ($\frac{L}{W}$)

Due to the complexity of our experimental system, we can only estimate through a mathematical expression the time-evolution of the geometrical factor ($\frac{\mathrm{L}}{\mathrm{W}}$). L and W are the superficial dimensions of the sample, length and width, respectively. Our experimental system measures the electrical resistance of the sample, but expressions (13), (14), (19), and (20) in the manuscript require to know the sample electrical resistivities. This fact makes necessary to determine the geometric factor ($\frac{\mathrm{L}}{\mathrm{W}}$). To this aim, we have measured the sample electrical resistance (R) with the four-point in-situ system and the resistivity (ρ) by the Van der Pauw method. Then we have:

$$\left(\frac{L}{W}\right) = \frac{R \cdot d}{\rho} \qquad \text{(SI 33)}$$

The above expression can now be applied to our four samples (samples B, C, D, and E), where R is the electrical resistance obtained in the *in-situ* system once the samples are cooled (i.e. room temperature, RT). The electrical resistivity, ρ, is measured at RT by the Van der Pauw method, and d is the thickness of the sample. We then adjust the obtained (L/W) by an expression whose behavior is similar to those published for simpler systems[9,10], resulting for stage 2 in:

$$\left(\frac{L}{W}\right) = \frac{-0.11}{1+exp\left(\frac{(t-0.11)}{0.03}\right)} +0.41 \qquad \text{(SI 34)}$$

The obtained trend is presented in Figure S10.

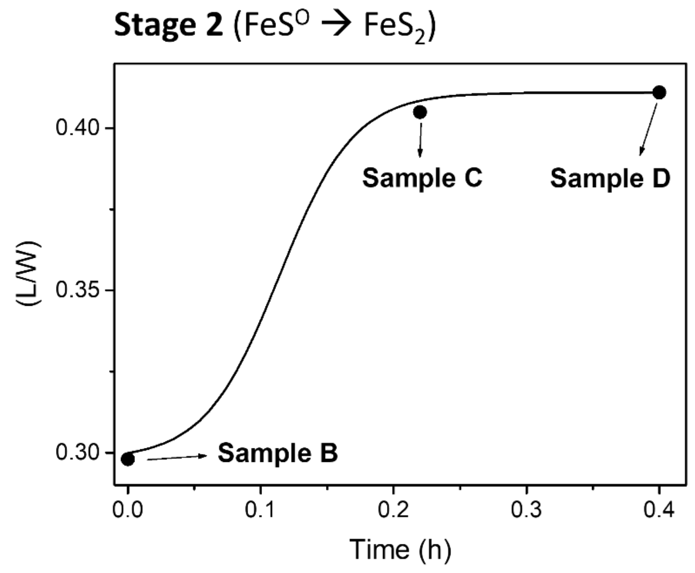

Figure S10. Time evolution of the geometric factor, (L/W), during stage 2. The dots indicate the experimental values from each one of the samples, and the continuous line represents the mathematical expression (SI34).

In the case of stage 3, the adjustment of an expression that correlates the geometrical factor values of samples D and E is more complex, as any function will adjust to a set of two points. However, the geometrical factor evolution has an almost negligible effect on the fitting. The thickness of the pyrite layer derived from the resistance measurements depends on the term $\frac{(L/W)(t)\cdot\rho^{h}}{R(t)}$. This means that the variations of the film thickness will be essentially correlated to the geometrical factor $(\frac{L}{W})$ and the resistance (assuming the hexagonal pyrrhotite resistivity constant). From D to E points, they vary by a factor of 1.3 and 15, respectively. Thus, the electrical resistance variation drives the film thickness evolution, not the geometrical factor. To prove this, we have considered three different functions, sigmoidal, linear, and exponential (see Figure S11a), adjusting the experimental data with them and obtaining equivalent results (Figure S11b).

Stage 3 ($FeS^h \rightarrow FeS_2$)

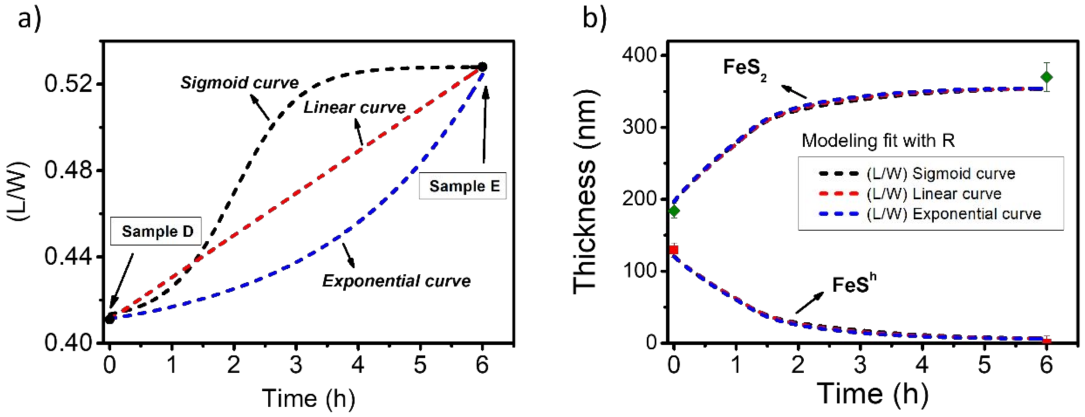

Figure S11. a) Time evolution of the geometric factor (L/W) during stage 3. The dots indicate the experimental values from each sample, and the dashed lines represent the sigmoidal (black), linear (red), and exponential (blue) mathematical expressions obtained from fitting the experimental points. b) Pyrite and hexagonal pyrrhotite thickness fitted by using the previous mathematical expressions for the geometrical factor.

Alternative modelling by considering diffusion as the limiting process during Stage 2 (Wagner diffusion)

Figure S12 shows the fit of the normalized electrical resistance, (R/R_o), and Seebeck coefficient (S) of the film during stage 2 ($Fe_{1-x}S^O \rightarrow FeS_2$). The experimental data are compared to the calculated ones when considering that the formation of pyrite in stage 2 is only controlled by Wagner diffusion, i.e., independent of S_2 partial pressure. A direct-eye comparison shows a poor agreement between experiment and fit.

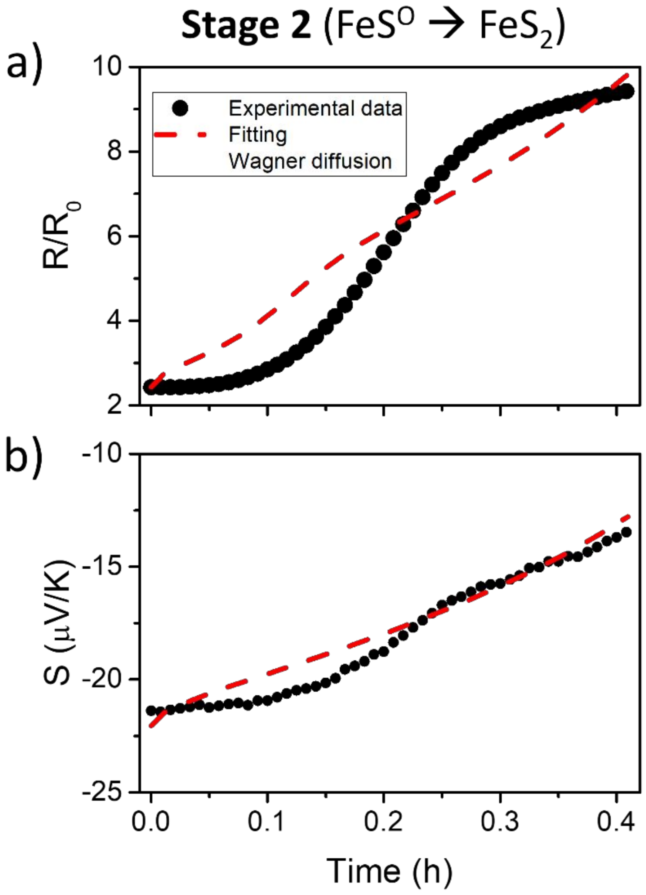

Figure S12. a) Electrical resistance (R) normalized to the initial value of Fe thin films (R_o) and b) Seebeck coefficient during Stage 2 of the process. The dots indicate the obtained experimental values, whereas dashed lines represent the theoretical values. These are obtained through expressions associated with a pyrite growth process limited by Wagner diffusion.

Alternative modeling by considering Wagner diffusion with constant diffusion coefficient as the limiting process during Stage 3

Figure S13a discusses the evolution of pyrite thickness during stage 3 in case that the kinetic of the sulfuration process is controlled not by the sulfur diffusion, but at the pyrrhotite/pyrite interface. In Figure S13b, the plot shows the deviation in pyrite thickness for t > 2.5h when considering a Wagner diffusion process with a constant diffusion coefficient. Figure S14 shows the estimated Seebeck coefficient of sample E compared to the experimental data of a different sample sulfurated under the same conditions.

Stage 3 ($FeS^h \rightarrow FeS_2$)

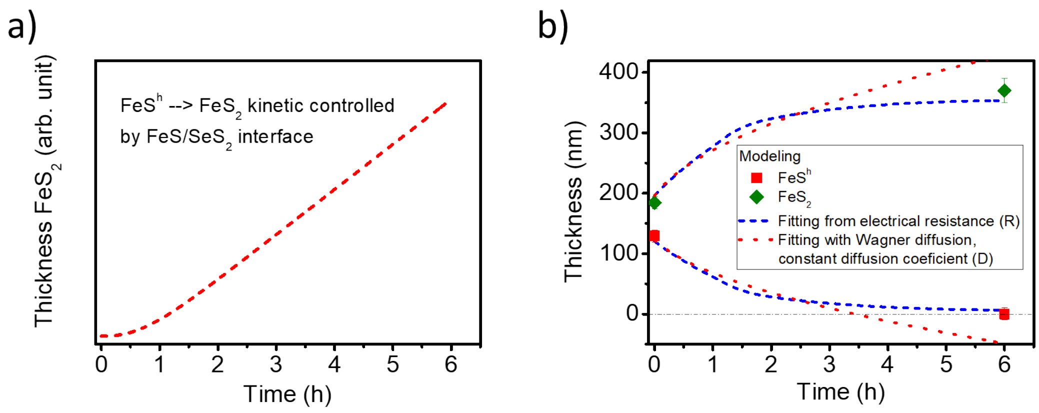

Figure S13. a) Estimated thickness evolution of pyrite film with time if the kinetic of Stage 3 is controlled by the pyrrhotite/pyrite interface b) Thickness evolution as fitted from the electric resistance (R) fitting (blue) and modeled by assuming a Wagner diffusion with constant diffusion coefficient as the limiting process (red). The symbols represent the estimated thickness of pyrrhotite (red) and pyrite (green) from the XRD diffraction pattern (Figure S2).

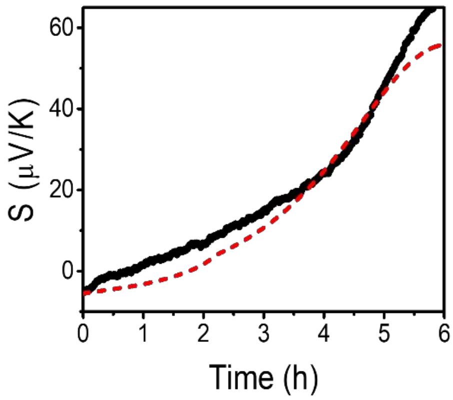

Figure S14. Estimated time evolution of the Seebeck coefficient of sample E (in red, dashed line) using the thickness calculated by the proposed kinetic model and compared to experimental data (continuous line) from a different sample sulfurated under virtually the same conditions.

References

(1) West, W. A.; Menzies, A. W. C. The Vapor Pressures of Sulphur between 100° and 550° with Related Thermal Data. *J. Phys. Chem.* **1929**, *33* (12), 1880–1892. https://doi.org/10.1021/j150306a002.

(2) Rau, H.; Kutty, T. R. N.; Guedes de Carvalho, J. R. F. High Temperature Saturated Vapour Pressure of Sulphur and the Estimation of Its Critical Quantities. *The Journal of Chemical Thermodynamics* **1973**, *5* (2), 291–302. https://doi.org/10.1016/S0021-9614(73)80089-8.

(3) Barnes, H. L.; Kullerud, G. Equilibria in Sulfur-Containing Aqueous Solutions, in the System Fe-S-0, and Their Correlation during Ore Deposition. *Economic Geology* **1961**, *56* (4), 648–688. https://doi.org/10.2113/gsecongeo.56.4.648.

(4) Pascual, A.; Yoda, S.; Barawi, M.; Clamagirand, J. M.; Ares, J. R.; Ferrer, I. J.; Sánchez, C. Iron Pyrite from Iron Thin Films: Identification of Intermediate Phases and Associated Conductivity-Type Transitions. *J. Phys. Chem. C* **2014**, *118* (46), 26440–26446. https://doi.org/10.1021/jp505303d.

(5) Morales, C.; Pascual, A.; Leinen, D.; Flores, E.; Muñoz-Cortes, E.; Leardini, F.; Ares, J. R.; Flege, J. I.; Soriano, L.; Ferrer, I. J.; Sanchez, C. Reaction Mechanism and Kinetic Model of Fe Thin Film Transformation into Monosulfides (FeS): First Step of the Fe Films Sulfuration Process into Pyrite. *J. Phys. Chem. C* **2022**, *126* (32), 13870–13883. https://doi.org/10.1021/acs.jpcc.2c02060.

(6) Keller-Besrest, F.; Collin, G. II. Structural Aspects of the α Transition in off-Stoichiometric Fe1−xS Crystals. *Journal of Solid State Chemistry* **1990**, *84* (2), 211–225. https://doi.org/10.1016/0022-4596(90)90320-W.

(7) King, H. E.; Prewitt, C. T. High-Pressure and High-Temperature Polymorphism of Iron Sulfide (FeS). *Acta Crystallogr B Struct Sci* **1982**, *38* (7), 1877–1887. https://doi.org/10.1107/S0567740882007523.

(8) Hirada, T. Magnetic and Electrical Properties of Iron SulfideSingle Crystals. *J. Sci. Hiroshima Univ. Ser. A* **1960**, *24* (2), 31.

(9) Yamashita, M. Geometrical Correction Factor for Resistivity of Semiconductors by the Square Four-Point Probe Method. *Jpn. J. Appl. Phys.* **1986**, *25* (Part 1, No. 4), 563–567. https://doi.org/10.1143/JJAP.25.563.

(10) Charge-Based and Probe Characterization. In *Semiconductor Material and Device Characterization*; John Wiley & Sons, Inc.: Hoboken, NJ, USA, 2005; pp 523–562. https://doi.org/10.1002/0471749095.ch9.
